# Optimization of Mass Spectrometry Imaging for Drug Metabolism and Distribution Studies in the Zebrafish Larvae Model: A Case Study with the Opioid Antagonist Naloxone

**DOI:** 10.3390/ijms241210076

**Published:** 2023-06-13

**Authors:** Yu Mi Park, Markus R. Meyer, Rolf Müller, Jennifer Herrmann

**Affiliations:** 1 Helmholtz Centre for Infection Research, Helmholtz Institute for Pharmaceutical Research Saarland (HIPS), Campus E8 1, Saarland University, 66123 Saarbrücken, Germany; yu-mi.park@helmholtz-hips.de; 2 Environmental Safety Group, Korea Institute of Science and Technology (KIST) Europe, 66123 Saarbrücken, Germany; 3 Department of Pharmacy, Saarland University, 66123 Saarbrücken, Germany; 4 Center for Molecular Signaling (PZMS), Institute of Experimental and Clinical Pharmacology and Toxicology, Department of Experimental and Clinical Toxicology, Saarland University, 66421 Homburg, Germany; m.r.meyer@mx.uni-saarland.de; 5 German Center for Infection Research (DZIF), 38124 Braunschweig, Germany

**Keywords:** zebrafish larvae model, drug metabolism and pharmacokinetics (DMPK), spatial drug distribution, mass spectrometry imaging (MSI), opioid antagonist, naloxone

## Abstract

Zebrafish (ZF; *Danio rerio*) larvae have emerged as a promising in vivo model in drug metabolism studies. Here, we set out to ready this model for integrated mass spectrometry imaging (MSI) to comprehensively study the spatial distribution of drugs and their metabolites inside ZF larvae. In our pilot study with the overall goal to improve MSI protocols for ZF larvae, we investigated the metabolism of the opioid antagonist naloxone. We confirmed that the metabolic modification of naloxone is in high accordance with metabolites detected in HepaRG cells, human biosamples, and other in vivo models. In particular, all three major human metabolites were detected at high abundance in the ZF larvae model. Next, the in vivo distribution of naloxone was investigated in three body sections of ZF larvae using LC-HRMS/MS showing that the opioid antagonist is mainly present in the head and body sections, as suspected from published human pharmacological data. Having optimized sample preparation procedures for MSI (i.e., embedding layer composition, cryosectioning, and matrix composition and spraying), we were able to record MS images of naloxone and its metabolites in ZF larvae, providing highly informative distributional images. In conclusion, we demonstrate that all major ADMET (absorption, distribution, metabolism, excretion, and toxicity) parameters, as part of in vivo pharmacokinetic studies, can be assessed in a simple and cost-effective ZF larvae model. Our established protocols for ZF larvae using naloxone are broadly applicable, particularly for MSI sample preparation, to various types of compounds, and they will help to predict and understand human metabolism and pharmacokinetics.

## 1. Introduction

Mass spectrometry imaging (MSI) is a state-of-the-art and label-free technology that allows investigating the localization and spatial distribution of diverse molecular species in one analysis in various biological specimens [1,2]. In general, MSI is an integrated approach offering the possibility to combine mass spectrometry (MS) (e.g., time of flight (TOF), Fourier transform ion cyclotron resonance (FT-ICR), and Orbitrap) to spatial images generated from different ionization equipment (e.g., matrix-assisted laser desorption ionization (MALDI), desorption electrospray ionization (DESI), and secondary ion mass spectrometry (SIMS)) to match specific needs for analyzing various types of samples [2,3,4]. In particular, visualizing the tissue distribution of a parent molecule and its metabolites has opened a new era for the characterization of the physiological and pathological properties of drugs. Thus, MSI has been increasingly used in preclinical studies [1,5,6,7] and illicit drug testing [8,9,10].

Zebrafish (*Danio rerio*; ZF) models are gaining popularity as an in vivo model organism in many research studies in developmental biology [11,12,13]; toxicology [14,15]; pharmacological properties of drugs, such as absorption, distribution, metabolism, and excretion (ADME) [16,17,18,19,20]; human diseases [21,22]; and neurobiology [23,24,25].

In our earlier studies [26,27], we demonstrated that MALDI-MS images are crucial for investigating the distribution of two new synthetic cannabinoids (SCs; 7′*N*-5F-ADB and 4F-MDMB-BINACA) inside the ZF larval body. Notably, the ZF larvae metabolism of the two studied SCs was found to be very similar to reported human and rodent metabolisms, and using various administration routes further helped to increase the number of detected metabolites. Combining routine metabolite identification studies with MSI analyses for deciphering the spatial in vivo distribution of investigated drugs in the ZF model is a promising tool with the potential to cover large parts of preclinical DMPK (drug metabolism and pharmacokinetics), typically used in rodent models [26,27,28,29,30]. However, preparation of ZF larval sections for MSI analysis is technically challenging, and the discrepancy in terms of metabolite detection between two representative analytical platforms that we have used, LC-HRMS/MS and MALDI-MSI, still requires further investigation. Furthermore, there are so far only few reports on MSI studies with ZF larvae, in particular in the context of drug distribution and metabolism [31,32], since MSI is so far more commonly used for tissues [1,33], tumors [34,35], rodent organs [3,36], and adult ZF [37,38].

**Figure 1:**
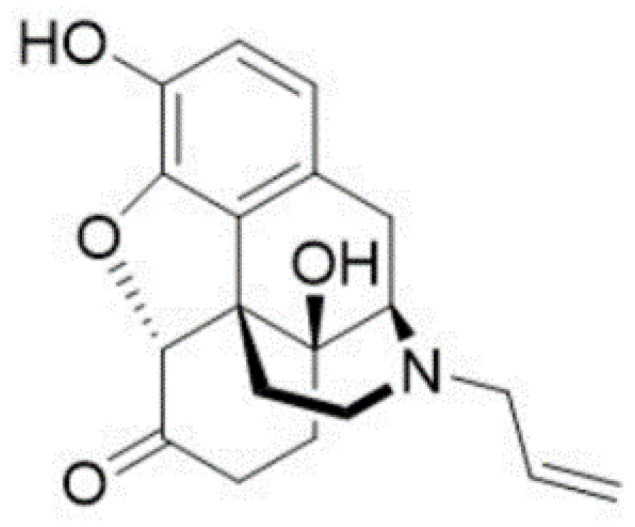
Chemical structure of naloxone.

In this study, we aimed to investigate the metabolism of the opioid antagonist naloxone (Figure 1) in ZF larvae and the common in vitro HepaRG model, as well as to compare our findings to already published data on humans [39,40,41,42,43,44,45,46,47,48] and other animal models [39,43,49,50,51,52]. Naloxone is widely used as an emergency therapy upon risk of opioid overdose, and it is distributed through a global take-home program [41,45,50,53]. We have chosen naloxone as a model drug, as it is known to cross the blood–brain barrier, and we expected to observe a distinct distribution pattern in the ZF larvae with a significant portion of the drug being accumulated in the brain region, as suggested by other studies [41,44,50,54]. Studying a drug with a distinct biodistribution pattern is considered a prerequisite to further optimize the MSI workflow in ZF larvae.

## 2. Results and Discussion

### 2.1. In Vivo Absorption, Metabolism, and Excretion Properties of Naloxone in the Zebrafish Larvae Model

In previous studies, we demonstrated that the administration routes into ZF larvae for two rather lipophilic synthetic cannabinoids, 7′*N*-5F-ADB [26,30] and 4F-MDMB-BINACA [27,29], have a significant impact on the observed metabolite patterns. For these substrates, direct administration via microinjection into different vital organs (i.e., heart ventricle, caudal vein, hindbrain, and yolk sac) was beneficial compared to conventional waterborne exposure. However, in this study, we mainly considered adding the water-soluble reference drug naloxone [45,50] to the embryo/larvae medium to treat ZF larvae in the course of in vivo ADME studies. Using this route for drug administration also significantly facilitates the MSI optimization procedures as proposed here, since it allows for higher throughput (in terms of overall sample numbers) compared to set-ups where microinjection into ZF larvae is required.

Following previously established workflows [26,27,28,29,30], the survival rates of ZF embryos and larvae after 5 d and 1 d treatment, respectively, were evaluated to determine a nontoxic concentration of naloxone that can be applied in subsequent in vivo ADME experiments. In parallel, the heartbeat of the exposed ZF larvae was measured at 5 days post-fertilization (dpf) as naloxone is described to induce cardiovascular events in humans (e.g., systolic blood pressure, respiratory stimulation, cardiovascular instability, and pulmonary edema) [44,50]. As a result, survival rates of naloxone-treated ZF larvae were 100% at maximum concentrations of 100 µM for 5 d exposure and 500 µM (highest assay concentration) for 1 d exposure. In addition, we did not detect significant effects on the heartbeat rates of ZF larvae treated with nonlethal concentrations, and we only found a slightly reduced heartbeat rate for ZF larvae treated for 1 d with 500 µM naloxone (Table S1). Thus, a concentration of 300 µM naloxone (1 d treatment of 4 dpf ZF larvae) was chosen for the subsequent metabolism studies.

The metabolite data of naloxone from the HepaRG in vitro model and from ZF larvae investigated in this study are summarized in Table 1. The immortalized hepatic stem cell line HepaRG is well known as an alternative to primary human hepatocytes for studies on drug metabolism [29,30]. The published in vivo data from humans [39,40,41,42,43,44,45,46,47,48] and different animal models [39,43,49,50,51,52] were added for comparison. Moreover, naloxone and some metabolites detected in this study are also listed along with additional information on metabolism and biology in the Human Metabolome Database (HMDB, https://hmdb.ca/ (accessed on 1 June 2023)). The metabolite screening of naloxone was carried out as described in previous studies [26,30]. More detailed information on human data and data from animal models is provided in Table S2, which also contains the metabolic reactions and exact masses of the detected metabolites. The amount–time profiles for naloxone and its three major metabolites (M2, M4, and M21) from HepaRG cells are provided in Figure S1.

**Figure 2:**
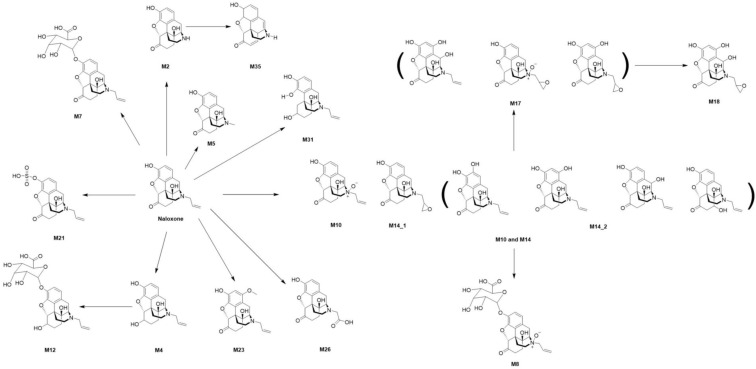
The reconstructed metabolic pathway of naloxone based on the metabolites detected in the ZF larvae model.

**Figure 3:**
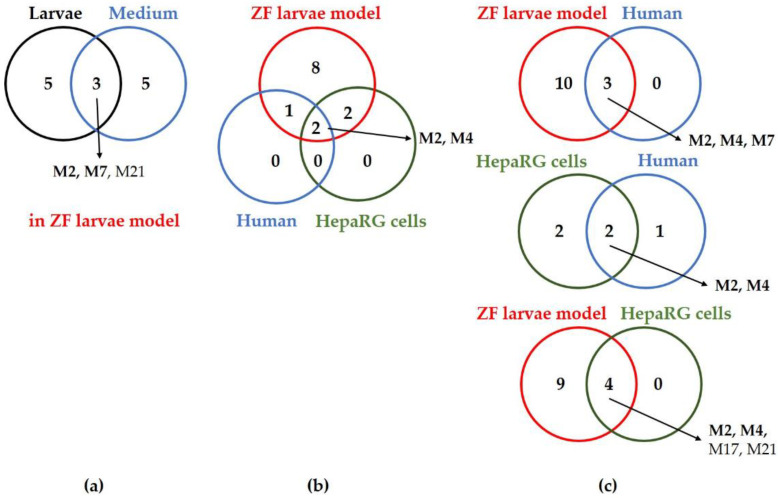
Comparison of the metabolites observed from three different models (human [39,40,41,42,43,44,45,46,47,48], ZF larvae, and HepaRG cells) using Venn diagrams: (**a**) thirteen metabolites were found in the ZF larvae model with three metabolites being detected in both the larvae and surrounding medium; (**b**) common metabolites detected in three different models; (**c**) their mutual comparability. Two metabolites (M2 and M4, in bold) were commonly found in all investigated models, and remarkably, the three most abundant human metabolites (M2, M4, and M7) were detected in the ZF larvae model.

Out of the 37 total theoretical metabolites of naloxone (see Table S2), three and seven metabolites were detected in human biosamples (plasma and urine) and rats, respectively (Table 1). Primarily, three metabolic reactions, *N*-dealkylation (M2), ketone reduction (M4), and glucuronidation (M7) are described in the literature on the human metabolism of naloxone, whereas the renal excretion of the glucuronic acid adduct is the major elimination route [41,45,50]. The other animal models (i.e., rabbit, chicken, and dog) produced two to three metabolites, whereas four metabolites were found in HepaRG cells. However, the structural isomers, M10 and M14, were counted as one metabolite because of the co-elution from the LC system used in this study. Notably, 13 metabolites in total were detected in the ZF model either extracted from the larvae or from the surrounding medium, whereas three metabolites (M2 (*N*-dealkylation), M7 (glucuronidation), and M21 (sulfation)) were detected in both types of ZF samples (Table 1 and Table S2). These findings highlight the superior performance of ZF larvae in metabolism studies, with a high number of possible metabolites being detected, which, in turn, greatly facilitates the reconstruction of naloxone metabolism pathways (Figure 2). In addition, the major metabolites from ZF larvae significantly overlap with those found in human and rodent models (Figure 3).

As detailed above, three metabolites, including the main human metabolite (M7), were found in both ZF larvae extracts and the surrounding medium (Figure 3a). In contrast, the commonly used in vitro HepaRG model did not reveal M7. However, two metabolites (M2 and M4) overlapped between human, ZF larvae, and HepaRG cells (Figure 3b). Encouragingly, metabolite data from the ZF larvae showed a 100% match rate in mutual comparability analyses with human data and with data from HepaRG cells (Figure 3c), and a match rate of 86% was determined when comparing ZF metabolite data to the rat model (Figure S2). Analyzing the mutual comparability on the basis of the rat model, we found 100%, 75%, and 46% match rates to humans, HepaRG cells, and ZF larvae, respectively (Figure S2).

Moreover, we assessed the uptake kinetics and biotransformation in the ZF larvae of naloxone over time through the semi-quantitative analysis of the relative peak intensities in order to explore the pharmacokinetic (PK) properties in ZF. From human PK, it is known that naloxone is rapidly metabolized with ca. 30 min and 60 min elimination half-times in human plasma and serum, respectively [41,44,50]. In ZF larvae, the uptake of naloxone increased steadily, reaching a maximum at 24 h exposure. Along with a steady increase in the amount of naloxone inside ZF larvae, the abundance of major metabolites, M7 and M21, dramatically increased from 6 h to 24 h of naloxone exposure, whereas M4 only slightly increased during the same observation period (Figure S3).

A detailed assessment of the composition ratio among the parent drug naloxone and these major metabolites inside ZF larvae is provided in Figure S4. It is worth mentioning that the major human metabolite M7 (38% after 24 h) was found in nearly equal amounts to naloxone (40% after 24 h) in the ZF larvae. One of five minor metabolites in the exposed larvae, M12 (glucuronidation of M4), showed a similar pattern in terms of the amount–time kinetics to the major metabolites, M7 and M21. The other four minor metabolites (M2, M8 (*N*-oxide in combination with glucuronidation), M23 (hydroxylation + methylation in benzene ring), and M35 (rearrangement after elimination of hydroxyl group of M2)) showed a slow incline of their production over the exposure time (Figure S5a). Consistent with the data from the ZF larvae and the most abundant internal metabolites, the major metabolite detected in the residual surrounding medium, M17 (dihydroxylation/hydroxylation in combination with epoxide formation/*N*-oxide in combination with epoxide formation) displayed a strong increase in terms of the relative amounts over time (Figure S5b). In contrast, the other external metabolites (M2, M5 (*N*-CH3; *N*-dealkylation), M10/M14 (*N*-oxidation/epoxide formation/hydroxylation in benzene ring), and M18 (dihydroxylation in combination with epoxide formation)) only slightly increased between 3 h and 24 h of exposure.

To investigate the elimination of naloxone in the ZF larvae model, the exposed larvae were transferred into compound-free medium at 28 °C after 1 h exposure to 300 µM naloxone. This time point was chosen since significant absorption and only minor conversion into metabolites of naloxone were observed in the uptake experiment (Figure S3). The internal peak intensity of naloxone showed an upward tendency until 10 min incubation in the drug-free medium (Figure S6), which could be explained by the balance of free fraction and bound naloxone. After the first 10 min, the amount of naloxone inside the ZF larvae declined until 6 h incubation (end of observation period) and, intriguingly, the amount of the most abundant two metabolites (M7 and M21) decreased as well. In contrast, the third most abundant metabolite, M4, that was analyzed in the previous uptake experiment was not quantified here as it was detected below a signal-to-noise ratio of 3. Comparing the composition ratio for naloxone and its major metabolites in ZF larvae upon transfer to the medium, the relative amount of naloxone was significantly reduced from 82% to 45% within 6 h, and in line with this, the relative amounts of M7 and M21 increased from 12% to 34% and from 7% to 21%, respectively (Figure S7). In summary, we can confirm that the data from the ZF larvae model on metabolism, absorption, and elimination of naloxone are well aligned and, thus, already covering three integral parts of the ADME experiments.

Taken together, in the ZF larvae model we observed fast circulation and efficient metabolism with a high similarity to human metabolism of naloxone as early as 10 min after compound exposure via conventional aqueous administration (Table 1 and Figure S3). In addition, the elimination of naloxone inside ZF larvae progressed further post 1 h continuous drug exposure (Figure S6), and a large portion of the initial naloxone amount in ZF larvae was excreted 6 h post-administration (Figure S7).

### 2.2. Localization of Naloxone and Its Metabolites in Whole-Body Sections of Zebrafish Larvae

**Figure 4:**
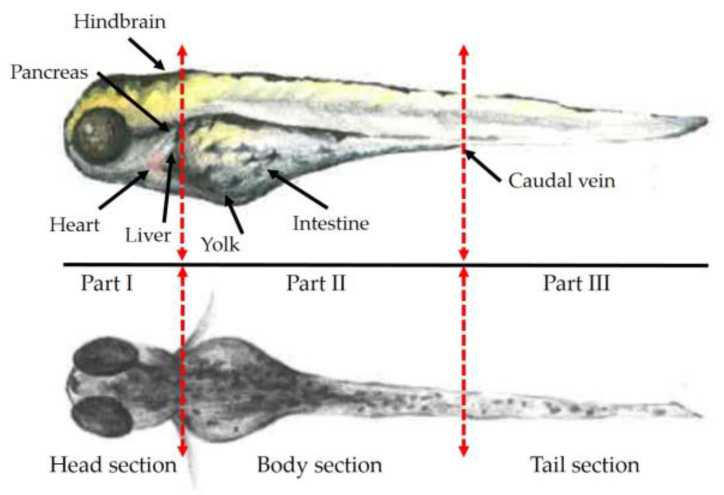
Prepared sections of ZF larvae at 5 days post-fertilization (dpf) in lateral ((**upper**) panel) and dorsal ((**lower**) panel) views. The head section included hindbrain, eyes, and heart. Yolk sac and other intestinal organs (e.g., liver, pancreas, and kidney) were part of the body section. The tail part did not contain any major organ compartments.

Opioids can act as receptor agonists leading to pharmacological effects, as described for the archetypical opioid morphine. Naloxone is used as an opioid antagonist due to a nonselective and competitive affinity to opioid receptors in the central nervous system (CNS), showing the highest affinity to the µ-opioid receptor (MOR) located in the brain, spinal cord, peripheral sensory neurons, and in the gastrointestinal tract [43,44,50]. In the present study, we examined the localization of naloxone and its metabolites inside ZF larval bodies initially using a simple dissection and extraction method. Naloxone-exposed larvae (300 µM, 1 h) were sectioned into three parts (head, body, and tail sections) using a surgical blade (Figure 4), with the goal of quantifying the levels of naloxone and its metabolites separately in these sections using methods as applied for the metabolism study detailed above. In order to obtain sufficient material for analyses, sections from 100 larvae were pooled for each individual sample. To account for differences in the total weight of the separated fractions undergoing extraction and analysis using LC-HRMS/MS, peak intensities were normalized to the determined total weight of each section sample.

**Figure 5:**
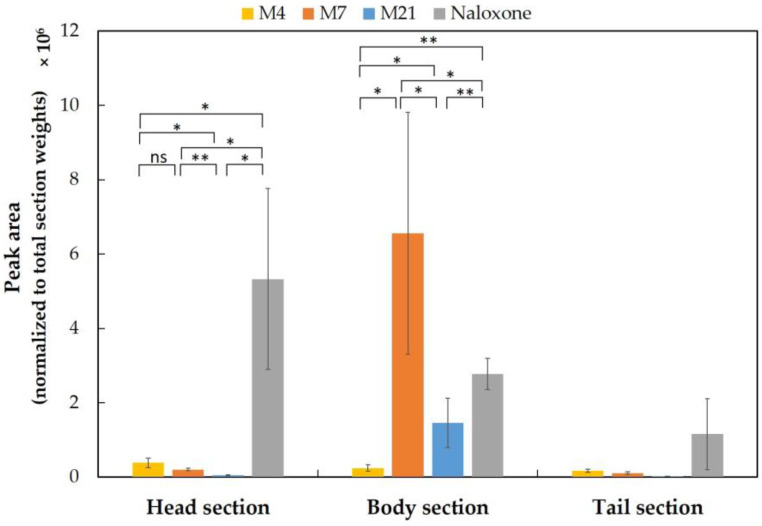
Sectional distributions of naloxone and its most abundant metabolites (M4, M7, and M21). The peak areas of individual compounds were normalized to the total weight of each section. Prior to sectioning, all ZF larvae were exposed to 300 µM naloxone for 1 h. The clustered columns are displayed as the mean ± standard deviation (n = 3), and the *p*-values between naloxone and three metabolites were calculated using one-way ANOVA (^ns^ *p* > 0.05, * *p* < 0.05, ** *p* < 0.01). All *p*-values were statistically significant in both the head and body sections, whereas the *p*-values for samples of the tail section could not be determined unambiguously because of the overall low-peak intensities.

Five metabolites (M2, M4, M7, M12, and M21), out of initially eight metabolites detected in whole-body ZF larvae, were detected using this experimental set-up. Other ZF metabolites of naloxone (M8, M23, and M35) were not detected in the ZF larvae sections because their production required ≥2 h exposure with the drug (Figure S5a). Indeed, naloxone was mainly localized in the head section, but it was also found in the body and tail sections, however, in lower amounts. In contrast, its most abundant metabolites, M7 and M21, were primarily found in the body section, whereas M4 was found in small amounts evenly distributed in all three prepared sections (Figure 5). The detection of two minor metabolites (M2 and M12) was consistent with the observed localization of M7 and M21 (Figure S8). Encouragingly, these initial results were fully in line with presumed in vivo drug and metabolite distributions, with naloxone having a high affinity to the µ-opioid receptor found at high expression levels in the brain and metabolites formed primarily in the liver. Thus, the data from whole-body sections served as excellent starting points for the envisaged optimization of our MSI methods to study the drug’s distribution in ZF larvae.

### 2.3. Optimization of MSI Methods to Study the Drug Distribution in Zebrafish Larvae—A Case Study Using Naloxone

Mass spectrometry imaging (MSI) provides a comprehensive method to study molecular distributions in a single experiment from biological specimens [1,2,3,25,55]. Over two decades, MALDI-MSI has been predominantly used, and in particular it is still gaining popularity in biomedical and cancer research [1,3,56,57]. Nevertheless, MSI is only rarely used for analyzing ZF larvae samples, which is probably because sample preparation from the tiny larvae poses significant technical challenges. In our previous studies [26,27], we were able to study the spatial distributions of two SCs, 7′*N*-5F-ADB and 4F-MDMB-BINACA, in ZF larval sections using MALDI-FT-ICR. These informative MS images supported our conclusions on how the route of drug administration impacts the in vivo distribution of a drug and its metabolism. However, there were some experimental challenges that still needed to be resolved prior to applying MSI for drug distribution studies in ZF larvae on a broader scale. In the current study, these issues were mainly related to discrepancies in MSI-based metabolite detection compared to routine LC-HRMS/MS measurements, which we attributed to the non-uniform formation of adduct ions in the MALDI-MSI set-up (cp. Table S3). In detail, we generally observed the presence of three adduct ions (proton, sodium, and potassium) of the parent compound for naloxone and the two SCs (7′*N*-5F-ADB and 4F-MDMB-BINACA) in the ZF larvae matrices. While evaluating the optimal embedding procedures and matrix deposition steps for MALDI-MSI of ZF larvae as detailed below, the predominant adduct formed of naloxone and its metabolites in the ZF homogenates was [M+K]^+^, and the [M+Na]^+^ species were found at a similar intensity under non-optimized conditions. This result for naloxone differed unexpectedly from the previous results, as 7′*N*-5F-ADB was found protonated and 4F-MDMB-BINACA was found as a sodium adduct in the MS images of ZF larvae [26,27]. We assumed that this finding might be caused by different ionization efficiencies of molecules in MSI dependent on the composition of the surrounding matrix [56,58,59,60,61]. However, it is crucial for relative spatial compound quantification by MSI that a single primary ion of the target species is formed predominately in order to maximize the detection limit (MDL) of a substrate and to correlate the compound abundance with MSI intensity plots.

Hence, further method optimization for MSI was performed to better fit the needs of ZF larvae analyses. As a first step, we used homogenates from naloxone-exposed ZF larvae to reduce sample variability and overcome issues originating from the sample inhomogeneity in the MSI of larval sections [56,58,59]. By spiking these ZF larval homogenates on the surface of a blank embedding medium layer, we examined the optimal conditions in terms of sample preparation by varying the composition of the lower-level embedding medium, cryosectioning, and matrix deposition steps for achieving more uniform ion detection patterns in MSI. Details on the studied conditions are summarized in Table S4. The listed parameters (i.e., steps) were assessed in sequential order.

**Figure 6:**
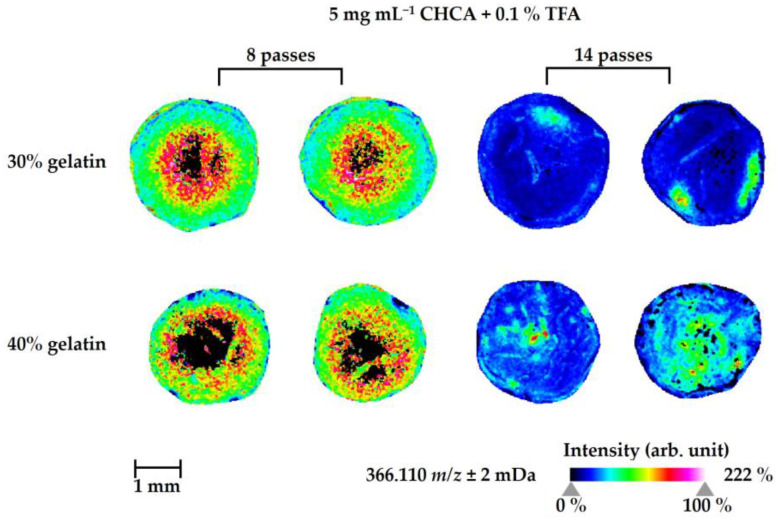
MALDI-MS images of the parent compound (naloxone, potassium adduct ion, *m*/*z* 366.110) in ZF larvae homogenates. The optimal matrix deposition conditions were determined as follows: 8 passes of spraying matrix deposition using 5 mg mL^−1^ α-cyano-4-hydroxycinnamic acid (CHCA) containing 0.1% trifluoroacetic acid (TFA) and 40% gelatin as the lower layer of the cryosectioned (10 µm) block. ZF larvae at 4 days post-fertilization (dpf) were treated by waterborne exposure with 300 µM naloxone at 28 °C for 1 d prior to homogenization with an ultrasonic homogenizer (pools of 30 larvae each). Each homogenate sample was prepared by spotting 1 µL of total homogenate onto a 30% and 40% gelatin layer, respectively. The MS images were prepared in duplicate. The images were generated by preparing a color map from blue (no detection) to purple (high local concentration), and they were further processed in 96 dpi resolution with 24-bit color under a weak denoising state.

In the MS images of naloxone from homogenates of treated ZF larvae (waterborne exposure, 1 d, and 300 µM), there were no specific distributional images when 2,5-dihydroxybenzoic acid (DHB) was used as a matrix, and naloxone was only detected—if at all—as a few random spots in the images using the matrix deposition conditions given in Table S4. It also has to be noted that the initial issue of different ion species negatively impacting the MSI of naloxone could not be resolved using DHB as a matrix reagent; the sodium and potassium ions of naloxone were present at similar abundances. In summary, 40% gelatin as an embedding medium with a higher concentration of DHB (30 mg mL^−1^) and 14 passes of spraying matrix deposition led to slightly better detection of naloxone compared to the other given conditions. Increasing the concentration of trifluoroacetic acid (TFA) in the DHB matrix solution led to slightly lower detectability; however, the differences were not significant. In contrast, when using α-cyano-4-hydroxycinnamic acid (CHCA) as a matrix, the naloxone detection significantly improved in both conditions using 30% and 40% gelatin as an embedding medium (Figure 6 and Figure S9). We determined eight passes of matrix spraying with 5 mg mL^−1^ CHCA solution containing 0.1% TFA as an optimal condition (Figure 6). Increasing the number of matrix spraying passes significantly reduced the intensity of naloxone, whereas a generally lower detection intensity was found when using the matrix at a higher concentration (15 mg mL^−1^ CHCA; Figure S9). Notably, in the CHCA matrix reagent, the potassium adduct ion was predominant in the MS images of the homogenates, whereas the sodium ion species was only observed in a few small spots. Overall, using sections of 40% gelatin for spotting the homogenates prior to matrix deposition led to better results with less vacant space of the measured homogenate than the 30% gelatin combined with a lower concentration of CHCA and higher spray deposition passes. In summary, the potassium adduct of naloxone ([M+K]^+^) was uniformly detected using the optimized procedure for the CHCA matrix deposition and, encouragingly, the relative abundance of naloxone as a [M+Na]^+^ species was significantly reduced. In particular, the utilization of ZF larval homogenates as a pre-step before establishing the optimal conditions for the MSI of the sectioned ZF larvae is convenient, and it diminishes many of the efforts required to accomplish MSI studies.

Moreover, under the optimized conditions for naloxone detection with MSI in the ZF larvae homogenates, three sections (head, body, and tail; Figure 4) of exposed ZF larvae were homogenized and then analyzed using MALDI-FT-ICR, and the results were compared to previously determined LC-HRMS/MS data. Consistent with the MSI results from whole-body homogenates, naloxone was mainly detected as a potassium adduct ion. In addition, we can confirm with MALDI-MSI of these sectioned homogenates that naloxone showed the highest abundance (on a qualitative scale) in the homogenate from the head region, whereas the amounts detected in the homogenate from the tail region were significantly lower (Figure S10).

Next, we attempted to quantify the relative naloxone amount in the MSI experiments for a fixed volume of homogenate (1 µL) using external calibration through spiking naloxone into blank ZF larvae homogenates (Figure S11). The calibration curves of naloxone were generated using linear (≤33 ng/µL) and logarithmic regression (>33 ng/µL) within different concentration ranges (Figure S12). The narrow range of linearity might be caused by matrix effects and ion suppression induced by the overall complex composition of the homogenates. This effect is generally considered a challenge in quantitative MSI studies [36,59,60,62], and it is assumed that it hinders the reproducible quantification of molecules in tissue sections through MSI. Thus, to produce reliable quantification data of a certain substance, an appropriate internal standard (ISTD) is required in order to compensate for matrix effects to the same degree as for the unlabeled investigated substance [36,59,60,61,62]. However, such a set-up is challenging to implement because of the lack and/or high price of custom-made ISTDs. Nevertheless, in future MSI studies, the use of an ISTD and additional washing steps by submersion of sections with organic solvent or buffer [4,55,63,64] prior to matrix deposition should be considered for more accurate quantification of absolute naloxone amounts.

**Figure 7:**
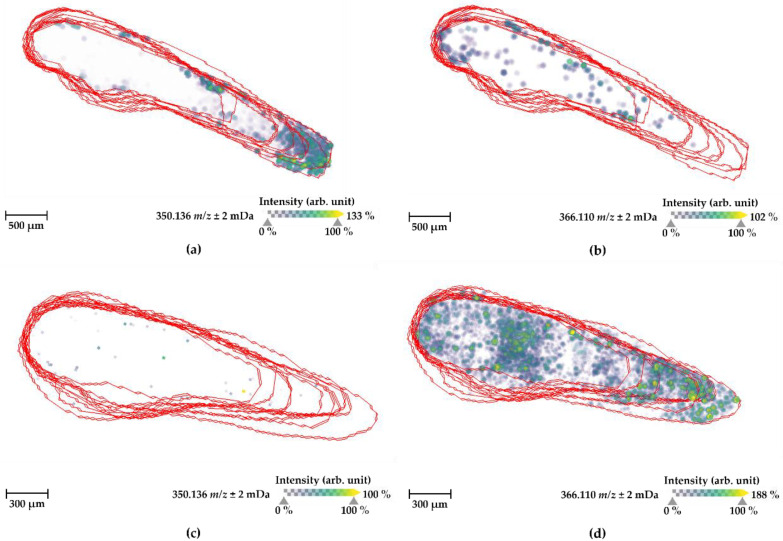
Comparison of the spatial distribution of naloxone (sodium adduct ion (*m*/*z* 350.136) and potassium adduct ion (*m*/*z* 366.110)) inside ZF larval whole body between (**a**,**b**) the protocol used in the previous studies [26,27] and (**c**,**d**) the optimal condition investigated in this study. All MS images were visualized with a three-dimensional view function in SCiLS Lab version 2022a Premium 3D software, based on the diagram of a ZF larva shown in Figure 4. The experimental conditions were the following: (**a**,**b**) 14 passes of spraying matrix deposition using 15 mg mL^−1^ DHB containing 0.1% TFA and 40% gelatin embedding medium, a total of 10 slides for one respective larva; (**c**,**d**) 8 passes of spraying matrix deposition using 5 mg mL^−1^ CHCA containing 0.1% TFA and 40% gelatin embedding medium, a total of 11 slides for one respective larva. The images were generated by preparing a color map from blue (no detection) to yellow (high local concentration) with a 50% transparency option, and then they were further processed in 96 dpi resolution with 24-bit color under a denoising state. The red lines indicate individual larval body sections that were overlapped for 3D visualization, and approximately 15 sections were usually utilized for this visualization.

Having determined an improved protocol for the detection of naloxone in ZF larvae homogenates, we set out to test these conditions for distributional MS images of naloxone inside ZF larvae whole-body sections (cp. Figure 4). We compared these MS images to those generated by our previous protocol (Figure 7) [26,27]. In addition, the intensity of the three adduct ions of naloxone was analyzed in the larval sections to confirm an enrichment of a single ion species under the optimized conditions (Figure S13). In particular, a 3D view function for MS images was used to look over the distribution of molecules inside the small-sized ZF larva and to verify their localizations in specific organs without any loss of detection information. Since one representative slide cannot explain an entire body distribution of the substrate with stereoscopic vision, this 3D function can reconstruct many cryosectioned larvae sections altogether into one entire larva MS image and also complement practical errors that occurred during embedding and cryosectioning processes. In practice, a sloping position of larva in an embedding medium occasionally occurs and then leads to partial or biased body sections.

In conclusion, optimizing the sample preparation and using 3D visualizations for MS images of naloxone (predominant detection as potassium ion) in ZF larvae significantly enhanced the intensity and detection frequency (Figure 7b,d). Interestingly, when comparing to the MS images generated under the previous non-optimized conditions (Figure 7a,c), several discrepancies were found, highlighting the need for compound and sample-specific optimization steps. In particular, the sodium adduct ion as major species was localized mostly in the tail part (Figure 7a), whereas the potassium ion was detected more or less randomly throughout the larval body at overall low abundance (Figure 7c). This distributional information, in turn, would have led to the conclusion that naloxone is accumulated in the tail of ZF larvae—a finding that can hardly be explained knowing that a significant proportion should be found particularly in the head region, as detected through LC-HRMS/MS-based analyses of ZF larvae described before. Having optimized the detection of naloxone in MSI, we were able to revise this false-positive finding and, indeed, analyzing the potassium adduct of naloxone under optimized conditions (Figure 7d) revealed a more reasonable distribution in view of the pharmacological properties of naloxone and our results from LC-HRMS/MS studies. In detail, naloxone was found at an overall high abundance with accumulation in the head part of the ZF larvae (in line with its property to cross the blood–brain barrier and high-affinity binding to the µ-opioid receptor) and at other accumulation spots in the main body part, where major organs are located, and in the tail region, possibly reflecting drug distribution processes. Overall, these findings highlight how appropriate sample preparation plays a vital role in MSI studies and emphasizes the necessity of finding the best conditions for analyzing a specific substrate.

**Figure 8:**
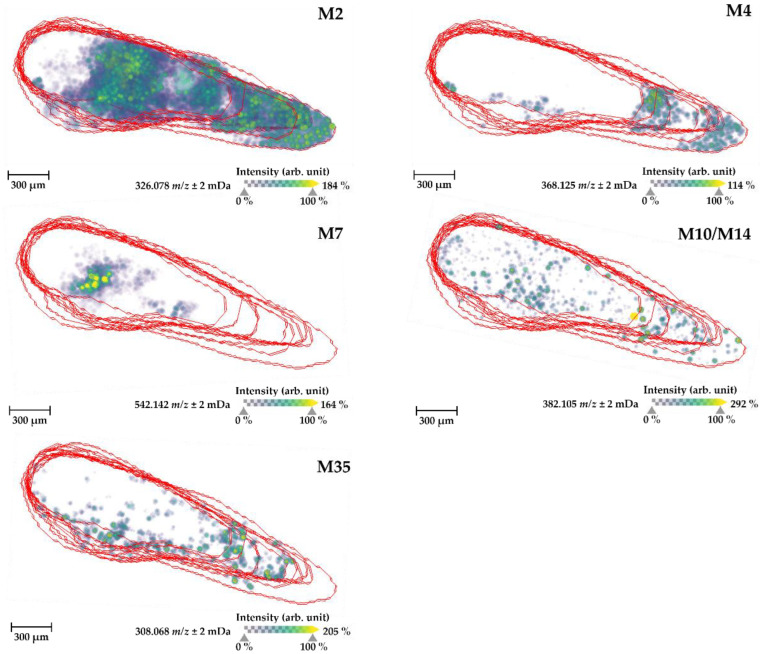
MALDI-MS images of the most abundant ZF metabolites of naloxone (M2 (*m*/*z* 326.078), M4 (*m*/*z* 368.125), M7 (*m*/*z* 542.142), M10/M14 (*m*/*z* 382.105), and M35 (*m*/*z* 308.068) potassium adduct ions) detected inside ZF larvae (whole body) prepared under the optimal conditions, as described above. All MS images were visualized with a three-dimensional view function in SCiLS Lab version Premium 2022a 3D software, based on the diagram of a ZF larva shown in Figure 4. The images were generated by preparing a color map from blue (no detection) to yellow (high local concentration) with a 50% transparency option and then were further processed in 96 dpi resolution with 24-bit color under a denoising state. The red lines indicate individual larval body sections that were overlapped for 3D visualization, and approximately 15 sections were usually utilized for this visualization.

Encouragingly, the overall inorganic salt adduct formation ([M+K]^+^ and [M+Na]^+^) of naloxone in the larvae sections prepared according to the newly developed protocol was approximately ten times higher than that observed in the MS images prepared according to the previously used method. Remarkably, through applying the optimized protocol, most naloxone signals derived from the highly abundant potassium adducted ion, with only very little contribution from the protonated species and the sodium adduct (Figure S13). In addition to the parent drug (Figure 7), five metabolites (M2, M4, M7, M10/M14, and M35) could also be visualized using MSI of the ZF larvae whole-body sections (Figure 8). M2 was strongly detected in all areas of the ZF larval body, except for the front head region, and M4 and M35 were distributed more in the peripheral and yolk sac sections. Interestingly, glucuronidated naloxone, M7, which is the primary human metabolite, was uniquely detected in a specific region where the ZF liver and pancreas are located, and M10/M14 was evenly distributed in the ZF larval body. Furthermore, these distributional properties of naloxone and its metabolites complemented findings of the whole-body and sectional larvae samples analyzed by LC-HRMS/MS, as described in the previous results.

## 3. Materials and Methods

### 3.1. Chemicals and Other Materials

Naloxone, dimethyl sulfoxide (DMSO), tricaine (3-amino-benzoic acid ethyl ester), trifluoroacetic acid, gelatin from cold water fish skin, 2,5-dihydroxybenzoic acid (2,5-DHB), and α-cyano-4-hydroxycinnamic acid (CHCA) were obtained from Sigma-Aldrich (Taufkirchen, Germany). Methanol (LC-MS grade), acetonitrile (LC-MS grade), and formic acid (LC-MS grade) were from VWR (Darmstadt, Germany). NaCl, KCl, MgSO_4_, Ca(NO_3_)_2_, and HEPES were purchased from Carl Roth (Karlsruhe, Germany). Stock solutions of all standards were prepared in DMSO at a concentration of 10 mM, and the solutions were stored for a maximum of one month at −20 °C. The working solutions were freshly prepared prior to each experiment. Six-well plates were obtained from Sarstedt (Nümbrecht, Germany). Differentiated HepaRG cells^®^ cryopreserved (HPR116), basal hepatic cell medium (MIL 600C), and thawing/plating/general purpose medium supplement (ADD 670C) were purchased from Biopredic International (Saint-Grégoire, France). A 24-well plate coated with type I collagen was obtained from Life Technologies GmbH (Darmstadt, Germany), and conductive indium-tin-oxide (ITO)-coated glass slides were obtained from Bruker Daltonics (Bremen, Germany). Zebrafish embryos of the AB wild-type line were initially obtained from the Luxembourg Center for Systems Biomedicine (Belvaux, Luxembourg). Dry, small granulate food was obtained from SDS Deutschland (Limburgerhof, Germany), and *Artemia* cysts (>230,000 nauplii per gram) were obtained from Coralsands (Wiesbaden, Germany).

### 3.2. ZF Maintenance and Embryo Collection

Zebrafish husbandry and all experiments with ZF larvae were executed according to EU Directive 2010/63/EU and the German Animal Welfare Act (§11 Abs. 1 TierSchG) in which all works were accomplished by following internal standard operating procedures (SOPs) based on published standard methods [65]. Adult ZF were kept in an automated aquatic ecosystem (PENTAIR, Apopka, UK), which is a continuous and real-time monitoring system under the following conditions: temperature (27 ± 0.5 °C), pH (7.0 ± 0.1), conductivity (800 ± 50 µS), and light–dark cycle (14 h/10 h). Fish were fed twice a day with dry, small granulate food and freshly hatched live Artemia cysts once per day. The ZF embryo/larvae medium (0.3× Danieau’s solution) was composed of 17 mM NaCl, 2 mM KCl, 0.12 mM MgSO_4_, 1.8 mM Ca(NO_3_)_2_, 1.5 mM HEPES, pH 7.1–7.3, and 1.2 µM methylene blue. To reproduce ZF embryos, the ZF pairs were kept overnight in standard mating cages, separated by gender. The following morning, the adult ZF started spawning as soon as the separators were removed. All fertilized eggs of ZF were collected and sorted using a Zeiss Stemi 508 stereo microscope (Carl Zeiss Microscopy GmbH, Jena, Germany). All embryos were raised in an incubator at 28 °C, with a daily medium change to clean the embryo cultures. The ZF larvae at 4 days post-fertilization (dpf) were used for drug metabolism studies.

### 3.3. Drug Treatment of ZF Larvae via Medium Exposure

The sample preparation following waterborne drug exposure is described elsewhere [26,27,28,29,55]. A nontoxic exposure concentration was chosen based on the survival rate as determined by in vivo maximum-tolerated concentration (MTC) experiments with 4 dpf (days post-fertilization) ZF larvae. To measure the heartbeat of the ZF larvae, the ZF larvae were anesthetized on ice and arranged. The heartbeat rates were determined using DanioScope software version 1.2.206 based on imaging using a stereo microscope (Zeiss Stemi 508 stereo microscope). For metabolite studies, 15 ZF larvae at 4 dpf were transferred to one well of a 6-well plate containing 3 mL of 0.3× Danieau’s medium with 300 µM naloxone. All exposure media contained a final concentration of 1% (*v*/*v*) DMSO, and the ZF larvae were treated for 24 h in an incubator at 28 °C. An additional 15 larvae were incubated in a compound-free medium containing only 1% (*v*/*v*) DMSO as a negative control (background masses). All treated larvae were rinsed twice with 1 mL of 0.3× Danieau’s solution prior to subsequent analyses.

### 3.4. Manual Sectioning of ZF Larvae

After exposure and washing, all larvae were transferred into a Petri dish using a pipette and then euthanized through cooling. Individual ZF larvae were aligned on a glass board for dissection. After the removal of excess medium around the larvae using a pipette (but not all to prohibit dryness of the sections and loss of sections), the immobilized larvae were cut into three sections (head, body, and tail sections; Figure 4) using a sterilized surgical disposable scalpel (B. Braun; Tuttlingen, Germany) under a microscope (Zeiss Stemi 508 stereo microscope). All sections were collected into pre-cooled tubes, and the excess medium was removed as much as possible with a pipette. Each section sample was prepared from a pool of 100 larvae in triplicate.

### 3.5. ZF Sample Preparation for Metabolite Analysis by LC-HRMS/MS

The 30 washed larvae treated with the procedures described above were pooled into a tube using a pipette and then euthanized by placing the tubes in ice water. After removing the excess medium, these larvae or the sectional larvae samples were snap-frozen in liquid nitrogen, followed by lyophilization for 4 h. The lyophilized larvae were stored for a maximum of one week at −20 °C before extraction. For metabolite identification, frozen larvae were thawed at room temperature for at least 30 min and extracted by vigorous vortexing for 2 min with 50 µL methanol. The sample was centrifuged at 10,000× *g* for 2 min at room temperature, and the supernatant was transferred to an autosampler vial. All pooled samples were prepared in triplicate. The extracts from ZF larvae were kept in a freezer at −20 °C and analyzed within one week after extraction.

The LC-HRMS/MS system was composed of a Dionex Ultimate 3000 RSLC system (Thermo Fisher Scientific, Germering, Germany) and maXis 4G HR-QTOF mass spectrometer (Bremen, Germany) with the Apollo II ESI source. The separation of the 5 µL aliquots from all larvae extracts was carried out using a linear gradient with 0.1% formic acid in water (*v*/*v*, eluent A) and 0.1% formic acid in acetonitrile (*v*/*v*, eluent B) at a flow rate of 600 µL/min. A Waters ACQUITY BEH C_18_ column (100 mm × 2.1 mm, 1.7 µm) equipped with a Waters VanGuard BEH C_18_ 1.7 µm guard column at 45 °C was used as the stationary phase. The linear gradient mode was programmed as follows: 0–0.5 min, 5% eluent B; 0.5–18.5 min, 5–95% eluent B; 18.5–20.5 min, 95% eluent B; 20.5–21 min, 95–5% eluent B; 21–22.5 min, 5% eluent B. Complementarily, UV spectra measurements were recorded using a diode array detector (DAD) in the range from 200 to 600 nm.

Mass spectra were recorded in the centroid mode ranging from 150 to 2500 *m*/*z* at a 2 Hz full-scan acquisition rate under auto MS/MS conditions in the positive ionization mode. External calibration was automatically performed by injecting sodium formate and calibration on the respective clusters formed in the ESI source before every LC-HRMS/MS run. All MS analyses were acquired in the presence of the *m*/*z* 622.0290, 922.0098, and 1221.9906 ions as the lock masses generated with the [M+H]^+^ ions of C_12_H_19_F_12_N_3_O_6_P_3_, C_18_H_19_O_6_N_3_P_3_F_2_, and C_24_H_19_F_36_N_3_O_6_P_3_. DataAnalysis software version 4.4 (Bruker Daltonics, Bremen, Germany) was used for the qualitative analysis.

With the limit of available reference standards, the expected naloxone metabolites were tentatively identified using MS/MS data (Figure S14). In addition, the relative quantification of the metabolites was conducted based on the peak areas under the assumption of similar ionization behaviors of the individual metabolites due to the lack of reference standards.

### 3.6. Mass Spectrometry Imaging Analysis of Naloxone and Its Metabolites by MALDI-FT-ICR

The ZF larvae treated using the procedures described in the SI were embedded in 30% and 40% (*w*/*v*) gelatin solution and then frozen and stored at −20 °C until cryosectioning. A single larva was cut with 10 µm thickness at −20 °C using a cryostat (MEV; SLEE, Mainz, Germany), and every section was put on a cold conductive indium-tin-oxide (ITO)-coated glass slide. In addition, 15 µm and 20 µm section thicknesses were also tested; however, this was not successful because of the coarse quality of the slides and the rolling up/solidification at the rim of the slides. All sections were scanned individually under a microscope for the alignment of the optical image of the sample in MALDI. These serial sections from one larva on one glass slide were deposited using a TM-Sprayer (HTX M5; HTX Technologies, Chapel Hill, NC, USA) with 2,5-dihydroxybenzoic acid (2,5-DHB; 15 mg mL^−1^ and 30 mg mL^−1^) and α-cyano-4-hydroxycinnamic acid (CHCA; 5 mg mL^−1^ and 15 mg mL^−1^) in acetonitrile:water (9:1, *v*/*v*) solution containing trifluoroacetic acid (TFA; 0.1%, 0.5%, and 1.0%), as described in Table S4, and then dried in a vacuum desiccator for ≥2 h. The spray nozzle temperature and flow rate of the pump were set up according to the manufacturer’s recommendations based on the chemical properties of each matrix reagent: 60 °C and 0.125 mL min^−1^ for DHB and 75 °C and 0.20 mL min^−1^ for CHCA. The dried glass slide was stored at −20 °C before the MALDI-FT-ICR measurements. In practice, as the detachment of the sections from the glass slide often occurred during the thawing step prior to the MALDI analysis, we recommend analyzing the samples within one week after the matrix deposition. This issue occurred mainly from the arid part of the sections embedded in both the 30% and 40% gelatin medium, and we assumed that it might be caused by the loss of water in the slides due to the prolonged storage.

For the MSI measurement, the sections of the ZF larvae were analyzed using MALDI and 7T SolariX FT-ICR (Bruker Daltonics, Bremen, Germany) in the positive ionization mode (*m*/*z* range 150–1000) using 40 laser shots per pixel with a raster width of 20 µm. Before acquiring the MALDI images, the mass calibration of the FT-ICR was performed with the calibration standard according to the manufacturer’s manual, and for auto-calibration of the MALDI of each laser measurement, the lock mass was set to *m*/*z* 273.0394 (for 2,5-DHB matrix) and *m*/*z* 212.0381 (for CHCA matrix). The MALDI-MSI data acquisitions and image data analyses in three dimensions were processed using ftmsControl version. 2.2.0, flexImaging version 5.0, and SCiLS Lab version 2022a Premium 3D software (Bruker Daltonics, Bremen, Germany).

### 3.7. ZF Larval Homogenate Preparation

The pooled 30 whole-body larvae and 100 sectional larvae samples were homogenized seven times for five seconds using an ultrasonic homogenizer (Zinnser Analytic GmbH; Frankfurt am Main, Germany) with 10 s intervals between the homogenization steps with 75% amplitude. The larval homogenate samples for the MSI measurements were prepared with spotting of 1 µL of total homogenate onto 30% and 40% gelatin and then dried in a vacuum desiccator for ≥1 h. To prepare the external calibration samples in the ZF larvae homogenate matrices, each calibration point was freshly prepared from a 5 mM stock solution, yielding final concentrations of 3, 10, 16, 33, 65, 98, and 164 ng per µL homogenate. The larvae homogenates for the calibration were pooled from 100 ZF larvae. All homogenates were scanned followed by a matrix deposition procedure, as described above.

### 3.8. In Vitro Metabolism Analyses Using HepaRG Cells

For further understanding of the naloxone metabolism in an in vitro model, differentiated HepaRG cells were investigated. According to the manufacturer’s instruction, the metabolism studies were implemented using adherent cells at 4 h after cell seeding (Biopredic International; Saint-Grégoire, France). All steps for the cell preparation were performed under sterile conditions. The differentiated cells frozen in a vial were thawed and seeded at a density of 4.8 × 10^5^ cells/cm^2^ in collagen-coated 24-well plates with 0.5 mL of thawing and seeding medium. These cell cultures were maintained at 37 °C in a humidified incubator (95% air humidity, 5% CO_2_). The thaw and seed medium was composed of MIL 600 (basal hepatic cell medium) supplemented by ADD 670C (thawing/plating/general purpose medium supplement with antibiotics) and pre-warmed to 37 °C before usage. For the cell exposure of naloxone, 150 µL aliquots of medium were removed from the 24-well cell culture plate, and the treatment was started by adding 150 µL of the naloxone-contained medium. These mediums were prepared by adding naloxone into the thaw and seed medium, yielding final concentrations of 100 µM with 0.2% (*v*/*v*) DMSO and 300 µM with 0.6% (*v*/*v*) DMSO in the total medium of one well.

Treatment was stopped after 0.1, 10, 30, 60, 120, 240, 360, and 1440 min, and the blank group (as a negative control) and two control groups (as a DMSO control; 0.2% and 0.6% (*v*/*v*)) were also prepared. All samples were prepared in duplicates. An aliquot of 30 µL supernatants of all medium was transferred into a tube, and for the extraction, 30 µL of cold acetonitrile containing with 0.1% formic acid was immediately added. The samples were vortexed and cooled in a freezer for 30 min at −20 °C. After centrifuging at 10,000× *g* for 2 min at 4 °C, 30 µL supernatant of the samples was transferred to an autosampler vial, and all samples were then dried in vacuo and resuspended with 30 µL of acetonitrile containing 0.1% formic acid. All incubation conditions were performed in duplicate. The analysis of these extracts and data acquisition were performed using the LC-HRMS/MS system conditions described in Section 3.5.

## 4. Conclusions

In this study, we aimed to improve MSI protocols in the context of metabolism and drug pharmacokinetics using the ZF larvae model as an alternative in vivo model for predicting human metabolism. Intriguingly, we demonstrated that the ZF model can reliably predict the human metabolism of naloxone; ZF larvae produced all three described main human metabolites along with ten additional ones, which were used to refine the metabolic pathways of the opioid receptor antagonist (Table 1 and Figure 2). In summary, while comparing the mutual similarity of the metabolisms, the ZF larvae showed a 100% match rate to both humans and the common in vitro HepaRG cells (Figure 3c). Moreover, ZF larvae showed an 86% match rate to rat, as a renowned in vivo model in pharmacology and toxicology (Figure S2). Moreover, the uptake and biotransformation kinetics of naloxone over time were assessed (Figures S3, S6, and S7).

The localization of naloxone inside the ZF larval bodies was first determined using manual sectioning of the larvae into the head, body, and tail regions, and we demonstrated through LC-HRMS/MS analyses that naloxone can be found in each body part with a certain accumulation in the head region (Figure 5). Given the pharmacological properties of naloxone as an opioid receptor antagonist, this result was fully conclusive and also consistent with reports on the morphine-like PK properties of naloxone in humans [43,44,50]. To optimize naloxone detection in MSI, the ZF larvae homogenates were utilized and we could optimize detection methods for naloxone distribution inside ZF larvae using the MSI approach (Figure 6 and Table S4), although FT-ICR-based quantification still needs further optimization in terms of reducing the matrix effects and availability of internal standards for reliable quantitative analyses (Figure S11). Importantly, we demonstrated that MSI of naloxone in ZF larvae under non-optimized conditions resulted in false-negative results with respect to the spatial distribution of the drug, a phenomenon which could be completely overcome when the newly developed sample preparation protocol was applied (Figure 7). Using the latter, we could not only reliably predict the in vivo distribution of naloxone, but we were also able to characterize the spatial distribution of major metabolites (M2, M4, M7, M10/M14, and M35; Figure 8). Furthermore, the discrepancy between MALDI-MSI and LC-HRMS/MS due to the formation of three different adduct ions—which was, indeed, the major rationale for undertaking the present study with naloxone—could be overcome, as we were able to implement protocols for MSI sample preparation that support the preferential formation of single ion species (Figure 7 and Figure S13).

Despite these encouraging results, some analytical and experimental challenges remain when using zebrafish larvae as a DMPK model, in particular studying the spatial distribution of metabolites via MALDI-MSI. For example, structural isomers of some naloxone metabolites could not be separated by the used LC-HRMS set-up, and it was in part challenging and time-consuming to spatially map such metabolites in MALDI-MS images despite having their high-resolution mass spectrum. Furthermore, up to now, only a relatively small number of drugs have been assessed in such zebrafish larvae models. This might be due to the fact that not all laboratories have routine access to the model and, in the case that waterborne exposure is not an ideal route for compound administration, microinjection needs to be applied, which requires some expert knowledge. Thus, further metabolism studies, particularly through MSI, are needed to fully understand the potential and usefulness of the zebrafish larvae model.

However, with the naloxone example we demonstrated that ZF larvae is a promising in vivo model, which can be broadly applied to study DMPK properties of a drug. In particular, drug metabolism in ZF larvae has been shown to reliably predict human metabolism, and we further consolidated this finding in the present study with naloxone. Having detailed information on the in vivo distribution of drugs and metabolites in ZF larvae would largely increase the impact of this model on routine screening and early preclinical profiling of investigational new drugs. In the present case study with naloxone, we were able to significantly improve MSI protocols providing reliable images of the in vivo distribution of naloxone. Importantly, the general workflows applied here for optimizing the MSI detection of naloxone inside ZF larvae is broadly applicable and will help to improve analytics of any other investigated molecule as part of DMPK studies in the ZF larvae model.

## Figures and Tables

**Table 1:** Summary of the detection patterns of naloxone and its metabolites from human biosamples, animal models, HepaRG cells, and zebrafish (ZF) larvae.

Compound	Human [39,40,41,42,43,44,45,46,47,48]		Rat [39,43,49,50,51]	Rabbit, Chicken [39]	Dog [43,52]	Data from This Study		
						HepaRG in Vitro Model ^†^	ZF Larvae ^‡^	
	Plasma	Urine	Plasma, Urine, Feces	Urine	Urine		Larvae	Medium
**Naloxone**	**+**	**+**	**+**	**+**	**+**	**+**	**+**	**+**
**M2**	**+**	**+**	**+**			**+**	**+**	**+**
**M4**	**+**	**+**	**+**	**+**	**+**	**+**	**+ ^c^**	
**M5**								**+ ^b^**
**M7**	**+**	**+**	**+**	**+**	**+**		**+**	**+**
**M8**							**+ ^c^**	
**M9**			**+**					
**M10**								**+ ^a,b^**
**M12**			**+**	**+ ^d^**			**+ ^c^**	
**M14**								**+ ^a,b^**
**M17**						**+ ^e^**		**+ ^b^**
**M18**								**+ ^b^**
**M21**			**+**			**+**	**+**	**+**
**M23**							**+ ^c^**	
**M26**								**+ ^b^**
**M35**			**+**				**+ ^c^**	
**Total number of detected metabolites**	**3**	**3**	**7**	**3**	**2**	**4**	**8**	**8**

All data from human samples and animal models were taken from published studies. Human plasma data were quoted from [42,46,47,48] and urine data from [39,40,41,42,43,44,45]. Rat plasma data were cited from [43,50,51] and urine and feces data from [42,43,49]. More details are shown in Table S2. ^†^ Concentrations of 100 µM and 300 µM naloxone were applied in the HepaRG in vitro model. ^‡^ ZF larvae were exposed to 300 µM naloxone. ^a^ The structural isomers M10 and M14, co-eluted in the LC-HRMS/MS set-up used in this study, and accordingly, these isomers were counted and quantified as one metabolite, although they derived from different metabolic reactions (see Figure 2). ^b^ The metabolites were only detected in the surrounding exposure medium and not from the extracted ZF larvae. ^c^ The metabolites were detected only in the extracted ZF larvae and not in the surrounding exposure medium. ^d^ The metabolite was not detected in rabbit. ^e^ The metabolite was only detected at the higher exposure concentration of 300 µM naloxone. + Peak detected. Metabolites M1, M3, M6, M11, M13, M15, M16, M19, M20, M22, M24, M25, M27–M34, M36, and M37 are not included in the table, as these were not detected in any of the listed models.

## Data Availability

Not applicable.

## Supplementary Materials

The supporting information can be downloaded at: https://www.mdpi.com/article/10.3390/ijms241210076/s1.
