# Supplementary material for: Optimization of Mass Spectrometry Imaging for Drug Metabolism and Distribution Studies in the Zebrafish Larvae Model: A Case Study with the Opioid Antagonist Naloxone
Source: Int J Mol Sci. 2023 Jun 13;24(12):10076. doi: 10.3390/ijms241210076 (PMC10298338; doi:10.3390/ijms241210076)
Supplement: Supplementary file 1 [file ijms-24-10076-s001.zip › ijms-2399585-supplementary.pdf]

Supplementary Materials

Optimization of Mass Spectrometry Imaging for Drug Metabolism and Distribution Studies in the Zebrafish Larvae Model: A Case Study with the Opioid Antagonist Naloxone

Yu Mi Park [1,2,3,†], Markus R. Meyer [4], Rolf Müller [1,3,5,*] and Jennifer Herrmann [1,5,*]

[1] Helmholtz Centre for Infection Research, Helmholtz Institute for Pharmaceutical Research Saarland (HIPS), Campus E8 1, Saarland University, 66123 Saarbrücken, Germany; yu-mi.park@helmholtz-hips.de
[2] Environmental Safety Group, Korea Institute of Science and Technology (KIST) Europe, 66123 Saarbrücken, Germany
[3] Department of Pharmacy, Saarland University, 66123 Saarbrücken, Germany
[4] Center for Molecular Signaling (PZMS), Institute of Experimental and Clinical Pharmacology and Toxicology, Department of Experimental and Clinical Toxicology, Saarland University, 66421 Homburg, Germany; m.r.meyer@mx.uni-saarland.de
[5] German Center for Infection Research (DZIF), 38124 Braunschweig, Germany
* Correspondence: rolf.mueller@helmholtz-hips.de (R.M.); jennifer.herrmann@helmholtz-hips.de (J.H.); Tel.: +49-681-98806-3000 (R.M.); +49-681-98806-3101 (J.H.)
† Current address: Division of Chemical Research, National Institute of Environmental Research, 42, Hwangyeong-ro, Seo-gu, Incheon 22689, Republic of Korea.

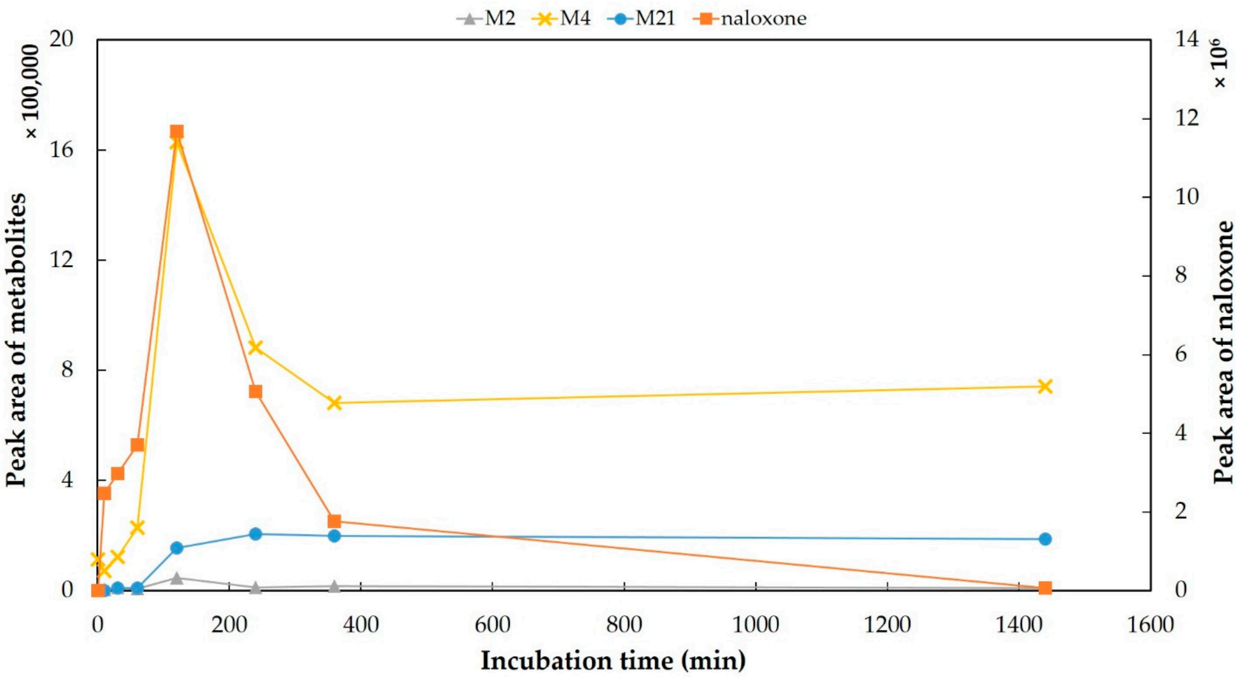

(a)

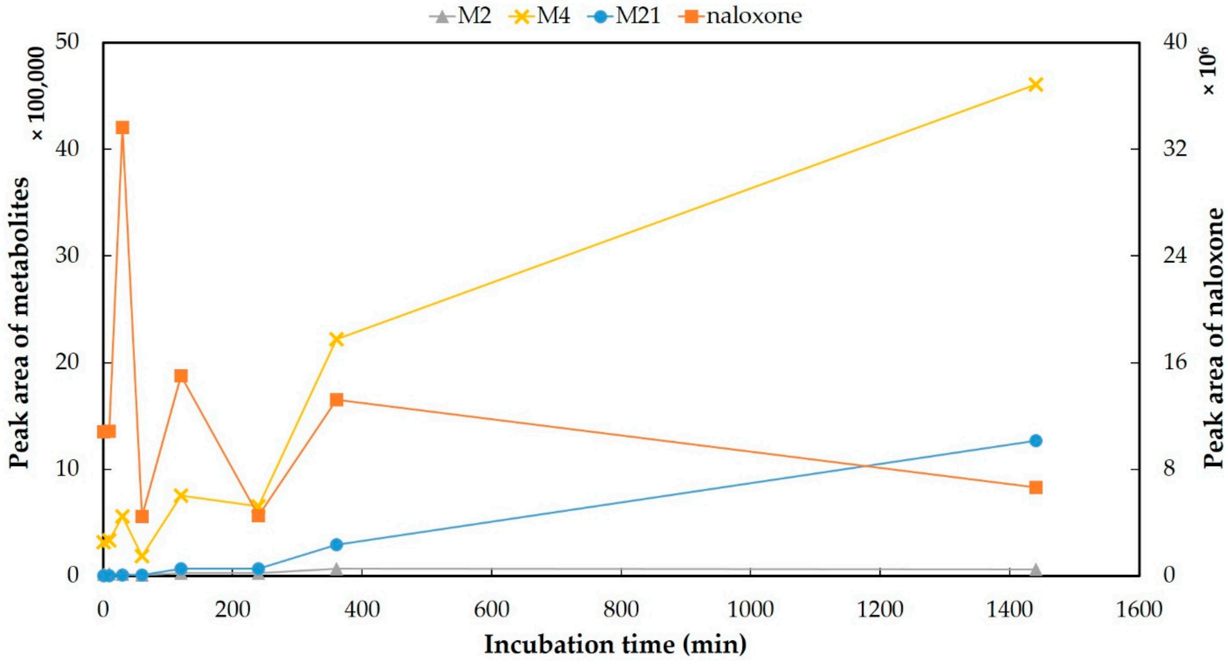

(b)

Figure S1. Internal amount-time profiles of naloxone and its main three metabolites (M2, M4, and M21) in HepaRG cells incubated with (a) 100 μM and (b) 300 μM naloxone, respectively. The cell medium was collected after 0.1, 10, 30, 60 120, 240, 360, 1,440 min of incubation ($n = 3$). The p-values between naloxone and these three metabolites at both concentrations were computed by one-way ANOVA, and all p-values represented significance at all incubation times, respectively ($p < 0.001$).

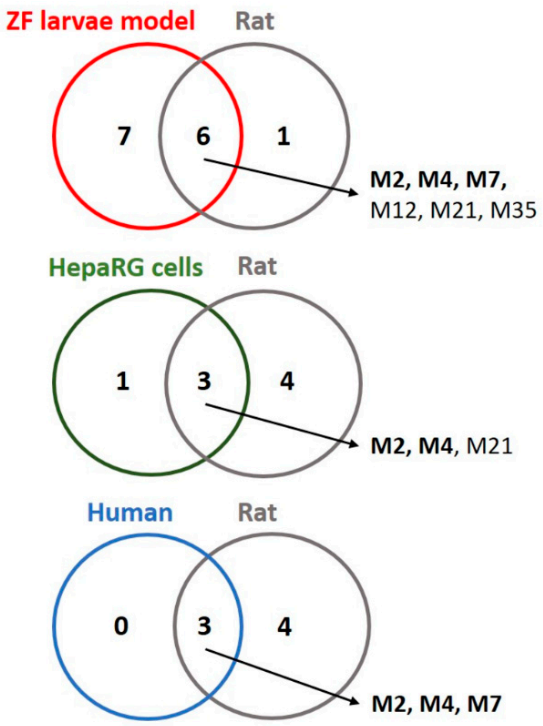

Figure S2. Mutual comparison of detected metabolites based on the rat model [1–5] with three different models (Zebrafish (ZF) larvae, HepaRG cells, and humans [1, 5–13]). Two metabolites (M2 and M4, in bold) were commonly detected in all investigated models, and the dominant three human metabolites (M2, M4, and M7) were found in both ZF larvae and the rat models.

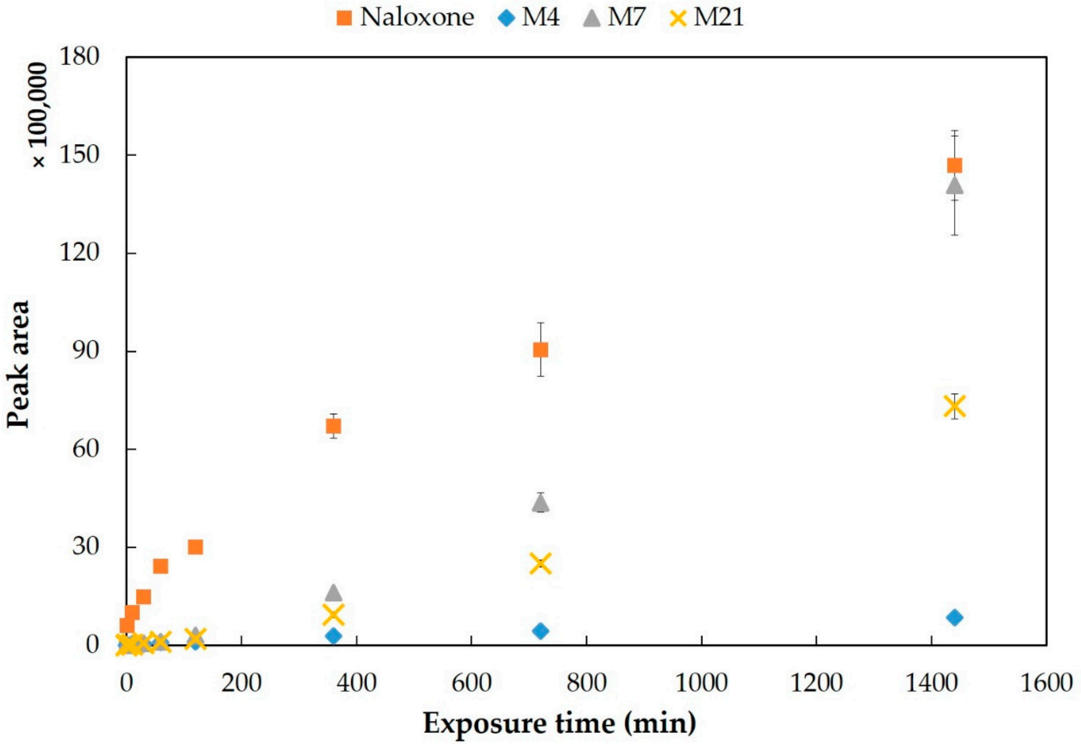

Figure S3. Internal amount−time profiles of naloxone and its most abundant metabolites (M4, M7, and M21) in ZF larvae exposed continuously with 300 μM naloxone. ZF larvae were exposed to the drug−containing medium for 0, 1, 10, 30, 60, 120, 360, 720, and 1,440 min, respectively ($n = 3$). All results from ZF larvae in this study are displayed as mean ± standard deviation from triplicates of 30 pooled larvae. The p-values between naloxone and its three abundant metabolites were computed by one-way ANOVA, and all p-values had statistical significance at all exposure times ($p < 0.001$).

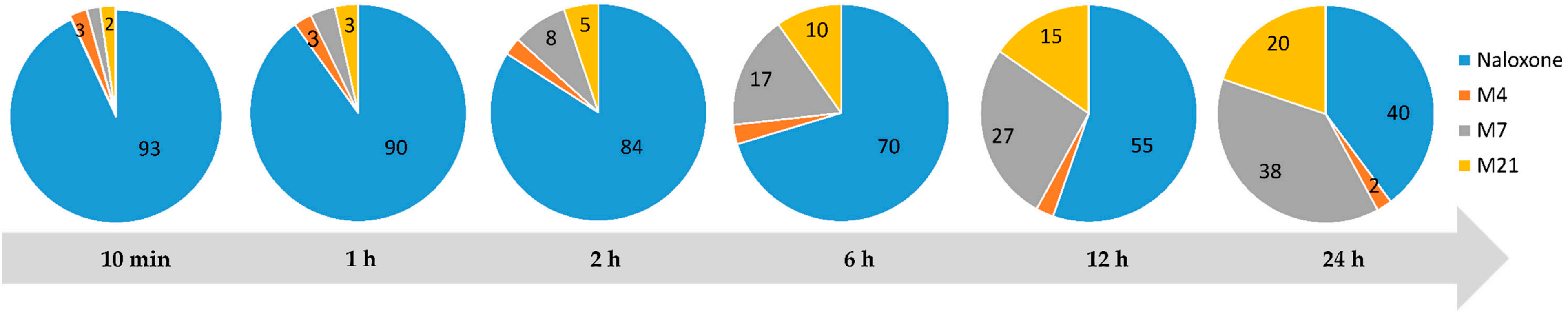

Figure S4. Composition pattern changes of internal naloxone and its major three metabolites (M4, M7, and M21) in ZF larvae after 10 min to 24 h exposure time of naloxone. All data are represented as mean of three replicates of 30 pooled larvae.

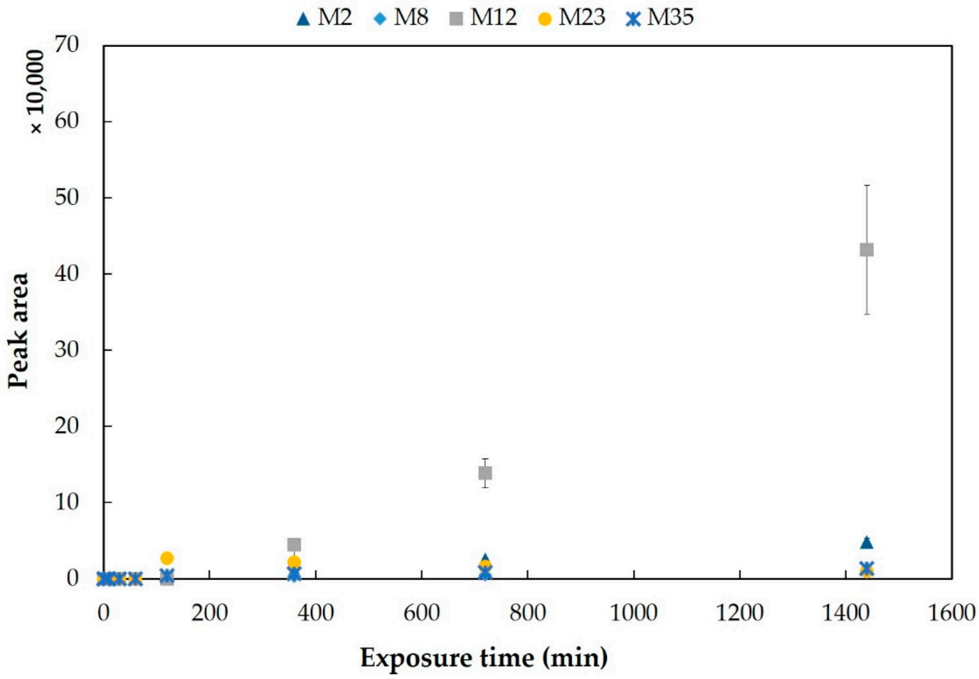

(a)

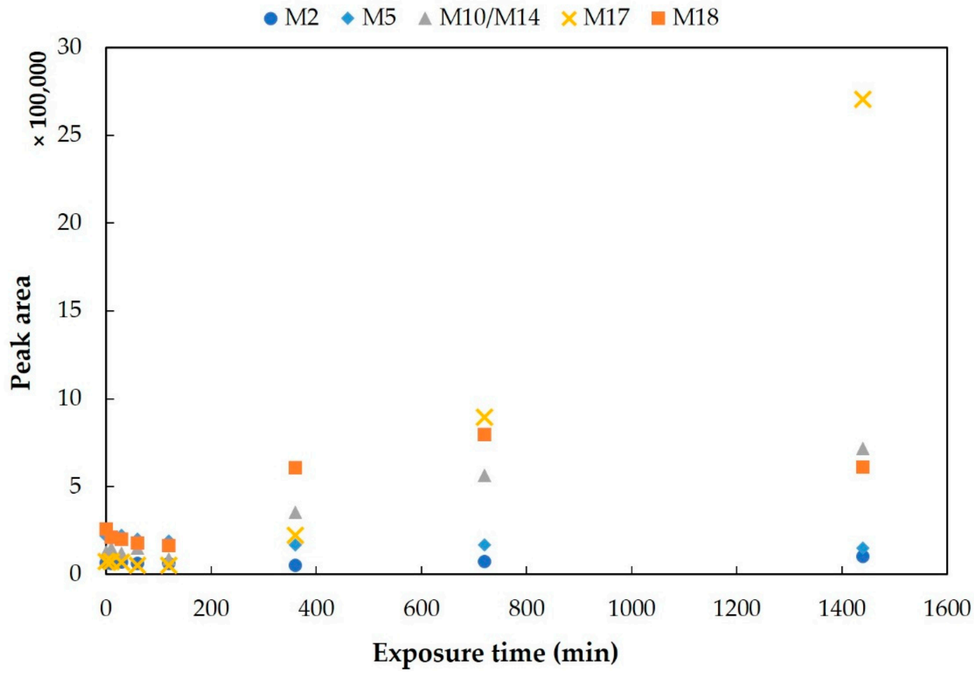

(b)

Figure S5. Internal amount-time profile of (a) five minor metabolites (M2, M8, M12, M23, and M35) in ZF larvae exposed to 300 µM naloxone and (b) detection of five metabolites (M2, M5, M10/M14, M17, and M18) in the surrounding medium after exposure of larvae to naloxone. ZF larvae were exposed to the drug-containing medium for 0, 1, 10, 30, 60, 120, 360, 720, and 1,440 min, respectively (n = 3). The residual medium is collected directly from each larvae sample at the given exposure time (n = 1). All results from ZF larvae in panel (a) are given as mean ± standard deviation from triplicates of 30 pooled larvae, and the p-values between naloxone and the respective five metabolites in the larvae were computed by one-way ANOVA. All p-values had statistical significance at all exposure times ($p < 0.001$).

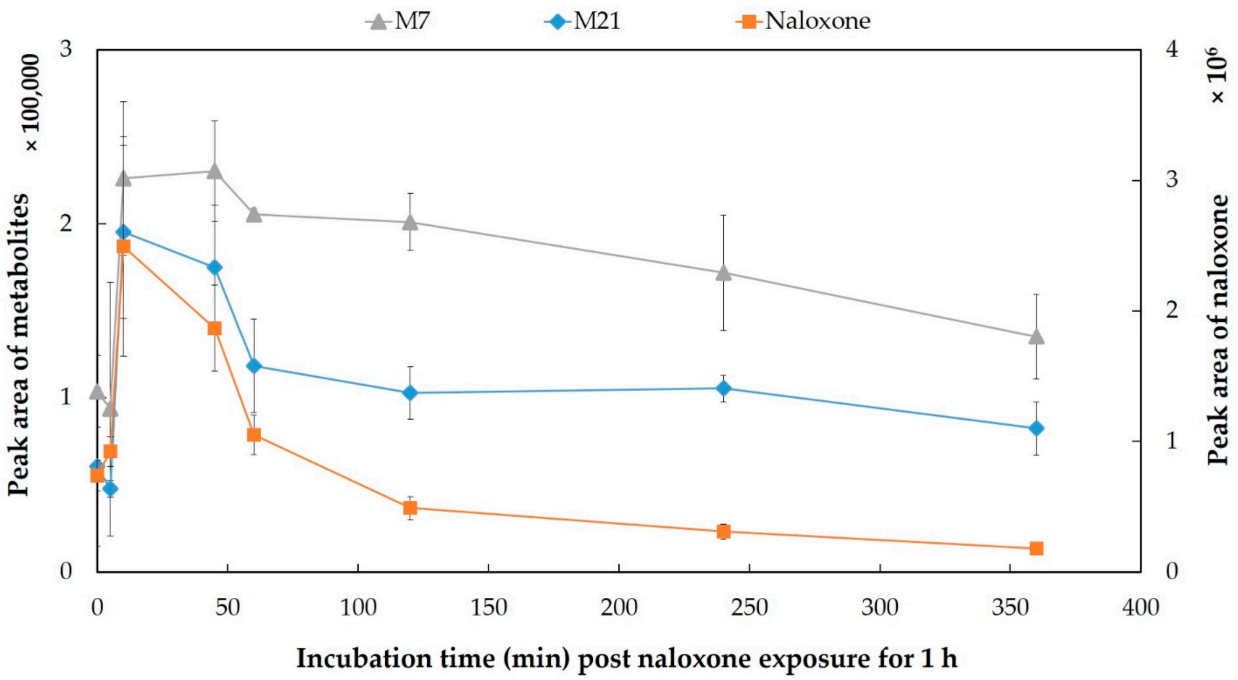

Figure S6. Elimination kinetics of naloxone and two abundant metabolites (M7 and M21) in ZF larvae following incubation in a compound-free medium post 1 h exposure with 300 μM naloxone. Incubations were performed at 0, 5, 10, 30, 45, 60, 120, 240, and 360 min ($n = 3$). All data are depicted by mean ± standard deviation, and the p-values between naloxone and the two other metabolites were calculated by one-way ANOVA. All p-values showed significance at all incubation times ($p < 0.001$).

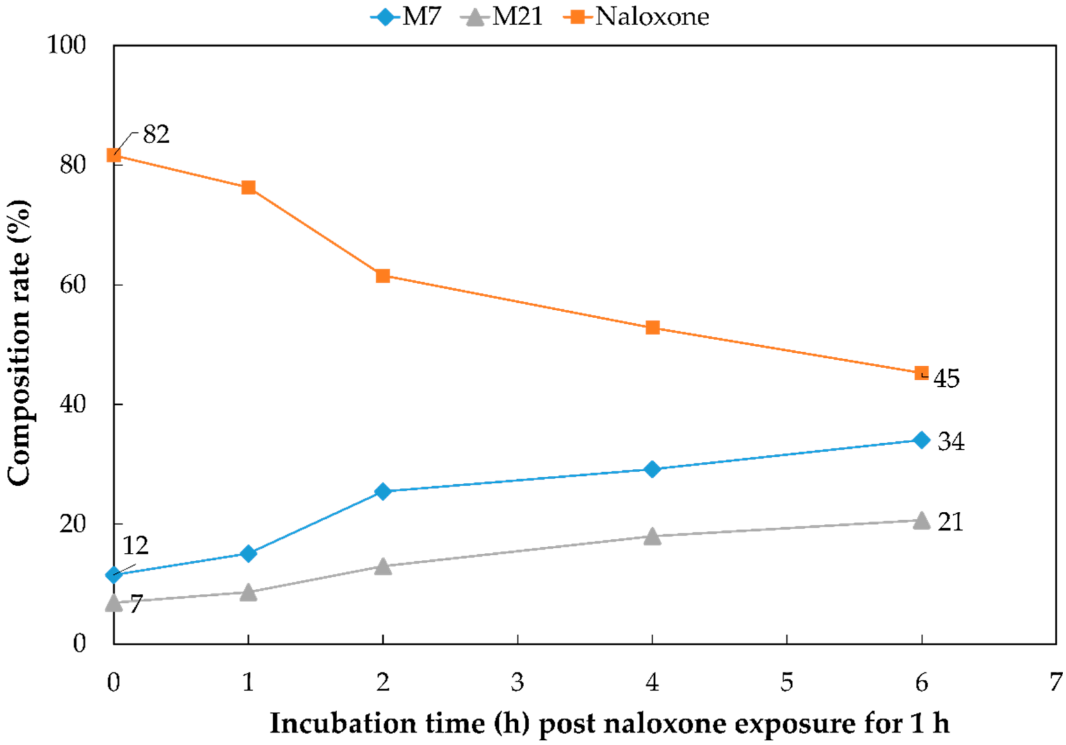

Figure S7. Composition rate changes of the relative amounts of naloxone and two major metabolites (M7 and M21) inside ZF larval bodies following incubation in a compound-free medium post 1 h exposure to 300 μM naloxone. Data were calculated on the basis of mean values given in Figure S6 ($n = 3$). All p-values between naloxone and these two metabolites were computed by one-way ANOVA and were statistically significant at all incubation times after 1 h naloxone exposure ($p < 0.001$).

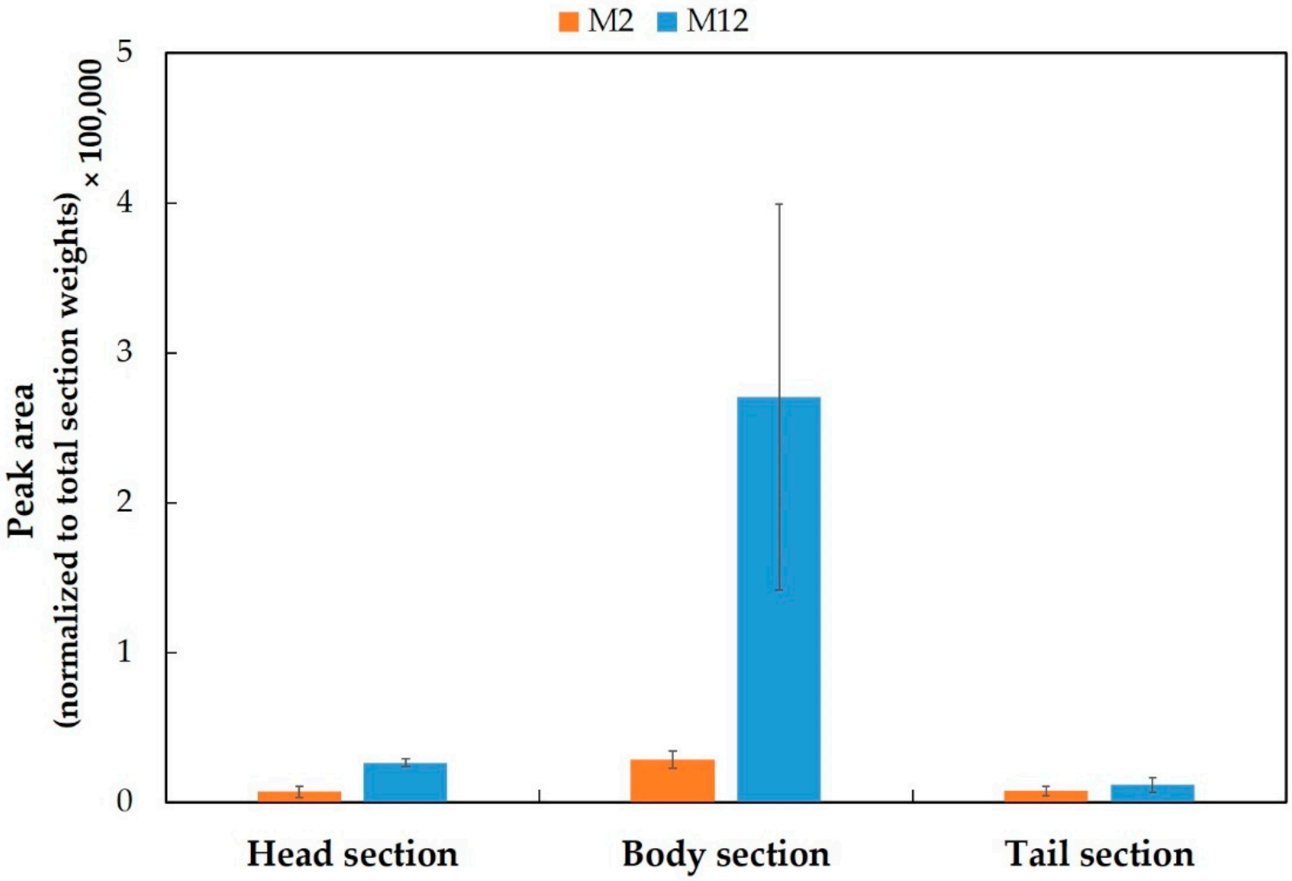

Figure S8. Sectional distributions of two minor metabolites (M2 and M12). The peak area of the substrate detected in the chromatogram was normalized individually with the total weight of each section, and the clustered columns are displayed as mean ± standard deviation (n = 3), and the p-values between naloxone and its two metabolites were calculated by one-way ANOVA ($^{*}p < 0.05$, $^{**}p < 0.01$). The p-values in the tail section could not be determined due to low intensity of M2 and M12.

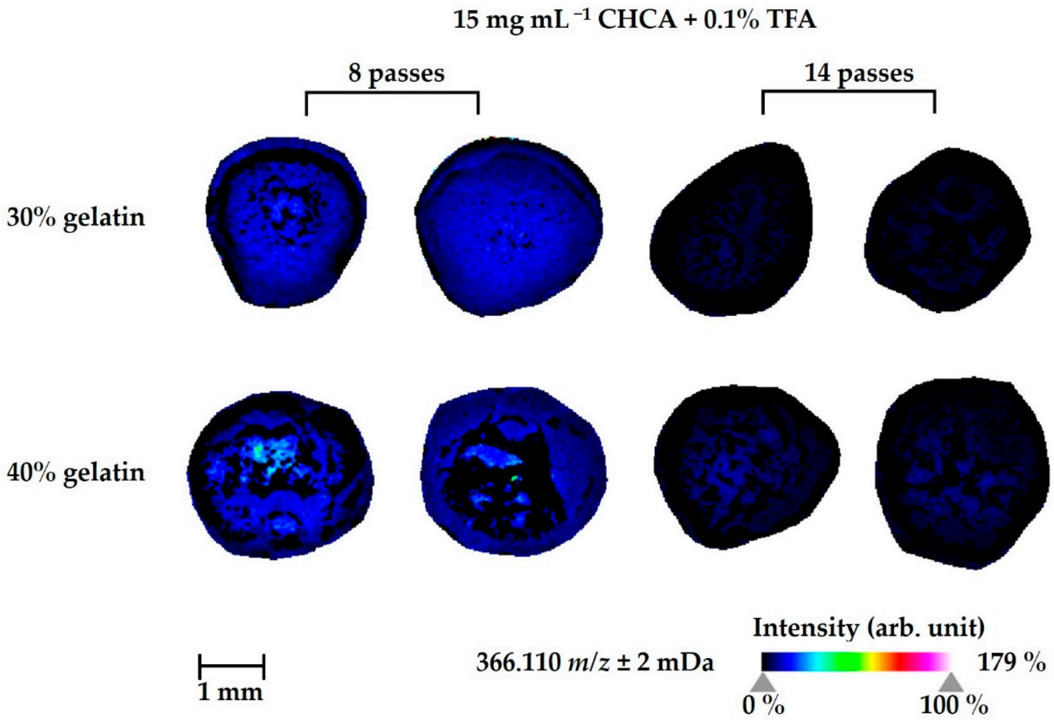

Figure S9. MALDI-MS images of the parent compound (naloxone, potassium adduct ion, *m*/*z* 366.110) in ZF larvae homogenates. The optimal matrix deposition condition was determined as follows: 8 passes of spraying matrix deposition using 15 mg mL^{-1} CHCA containing 0.1% TFA and 40% gelatin as lower layer of the cryosectioned (10 μm) block. ZF larvae at 4 days post-fertilization (dpf) were treated by waterborne exposure with 300 μM naloxone at 28 ˚C for 1 d prior to homogenization with an ultrasonic homogenizer (pools of 30 larvae each). Each homogenate sample was prepared by spotting 1 μL of total homogenate onto 30% and 40% gelatin layer, respectively. The MS images were prepared in duplicates. The images were generated by preparing a colormap from blue (no detection) to purple (high local concentration), and they were further processed in 96 dpi resolution with 24−bit color under a weak denoising state.

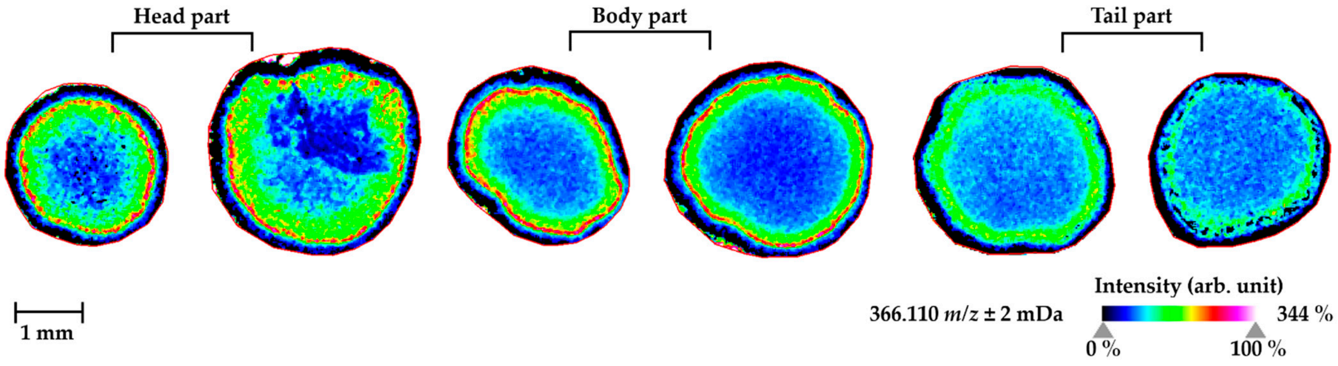

Figure S10. MALDI-MS images of the parent compound (naloxone, potassium adduct ion, *m*/*z* 366.110) in three sectional homogenates of ZF larvae (head, body, and tail region) after 1 d treatment of ZF larvae at 4 dpf by waterborne exposure with 300 μM naloxone at 28 °C prior to sectioning and homogenization. Each homogenate sample was prepared by spotting 1 μL of total homogenate onto 10 μm slices of 40% gelatin followed by the matrix deposition step as detailed in the main text (optimized condition). The duplicate MS images originate from individual pools of 100 larvae. The images were generated by preparing a colormap from blue (no detection) to purple (high local concentration) and then were further processed in 96 dpi resolution with 24-bit color under weak denoising state.

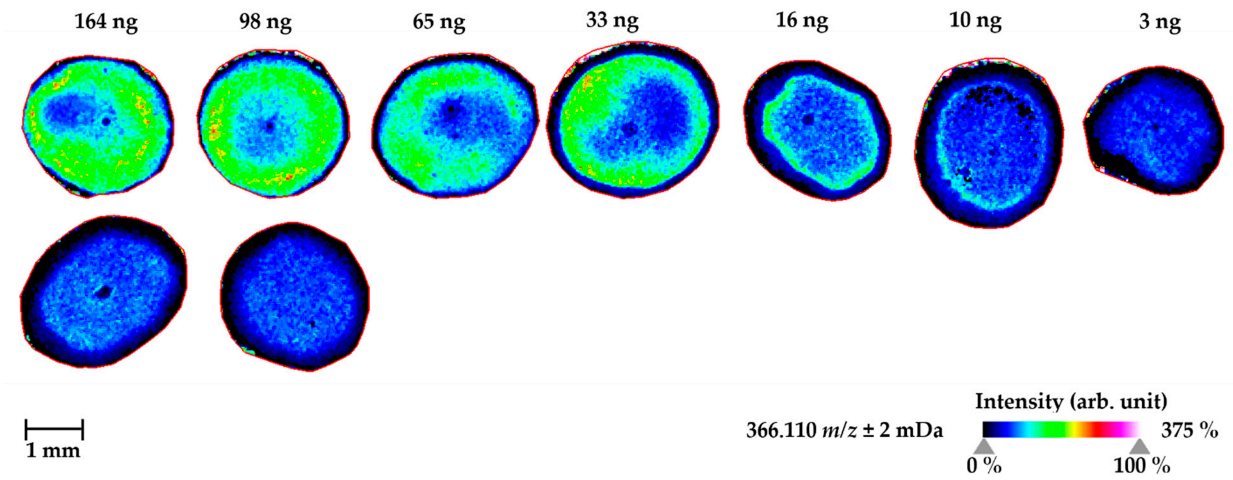

Figure S11. MALDI-MS images of naloxone (potassium adduct ion, *m*/*z* 366.110) at different concentrations in 1 μL of blank ZF larvae homogenates ((**upper**) panel) and equal amounts of homogenates from ZF larvae pre-exposed to naloxone ((**lower**) panel). ZF larvae at 4 dpf were treated by waterborne exposure with 300 μM naloxone at 28 °C for 1 d and then homogenized with an ultrasonic homogenizer. Each homogenate sample was prepared by spotting 1 μL of total homogenate onto a 40% gelatin layer as described above. All homogenates used originate from individual pools of 100 ZF larvae, and the exposed larvae homogenates were analyzed in duplicates. All these samples were prepared using the optimized conditions for MSI of naloxone in ZF larvae homogenates as described above. The images were generated by preparing a colormap from blue (no detection) to purple (high local concentration) and then were further processed in 96 dpi resolution with 24-bit color under weak denoising state.

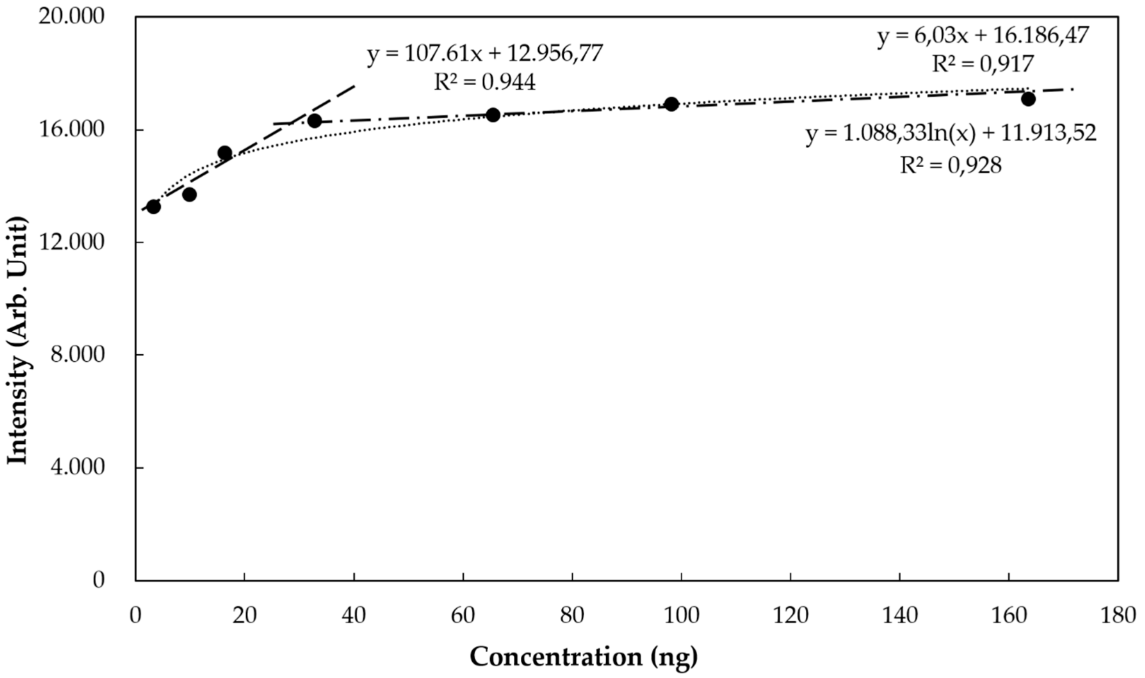

Figure S12. Calibration curves for naloxone (potassium adduct ion, *m*/*z* 366.110) spiked into ZF larvae homogenates at 3, 10, 16, 33, 65, 98, and 164 ng per 1 µL homogenate (n = 7). The obtained data were either analyzed by logarithmic regression (full concentration range; round dotted line; n=7) or by linear regression of different concentration ranges (3-33 ng, long dashed line and 33-164 ng, long dash-dot line; n = 4). The resulting calibration curves all showed a high degree of R-squared ($R^2 \geq 0.92$).

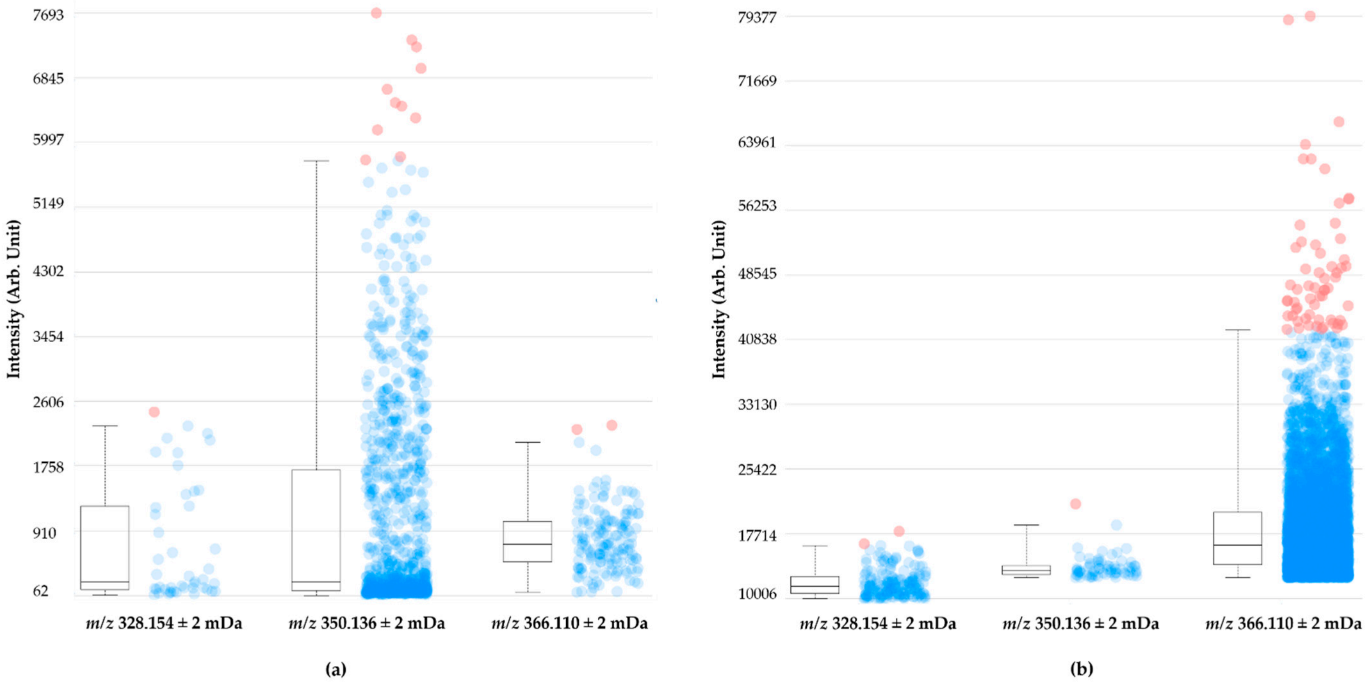

Figure S13. Comparison of the intensity of three naloxone adduct ions [proton (*m*/*z* 328.154), sodium (*m*/*z* 350.136), and potassium adduct ion (*m*/*z* 366.110)] in the MS images of drug-treated ZF larvae whole body sections visualized with a 3-dimensional view function in SCiLS Lab version 2022a Premium 3D software. ZF larvae samples were prepared according to (a) the protocol used in the previous studies [14] and (b) the optimized protocol developed as part of this study. In detail, the following conditions were used: (a) 40% gelatin embedding medium, 15 mg mL^{-1} DHB containing 0.1% TFA, 14 passes of matrix spraying, composed up of 10 slides from one respective larva; (b) 40% gelatin embedding medium, 5 mg mL^{-1} CHCA containing 0.1% TFA, 8 passes of matrix spraying, composed up of 11 slides from one respective larva. The intensity box part is depicted by the lower and upper boundaries representing the second and the third quartile, and the line extending vertically from the boxes indicates lower and upper quantiles whose relative values are set at 99% quantile. The cloud part shows how the intensity of the molecular image distributes in spectra of a sample. Blue dots represent the spectra between the lower and upper quantiles, and red dots represent outliers distributed outside these intensity intervals.

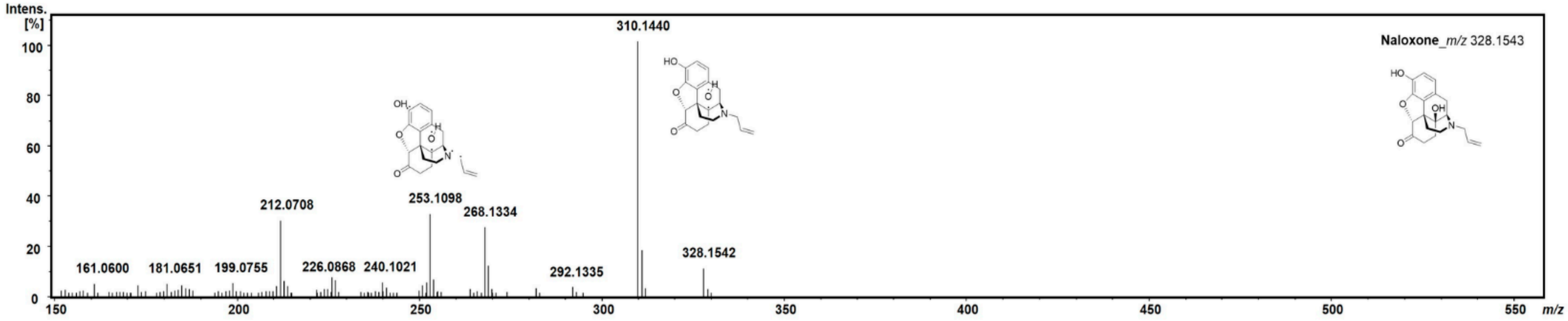

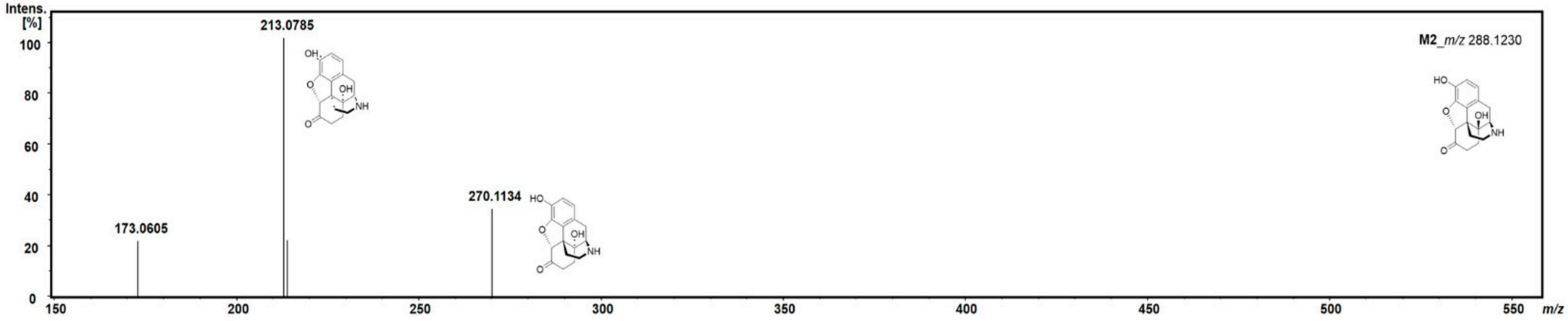

Figure S14. *Continued.*

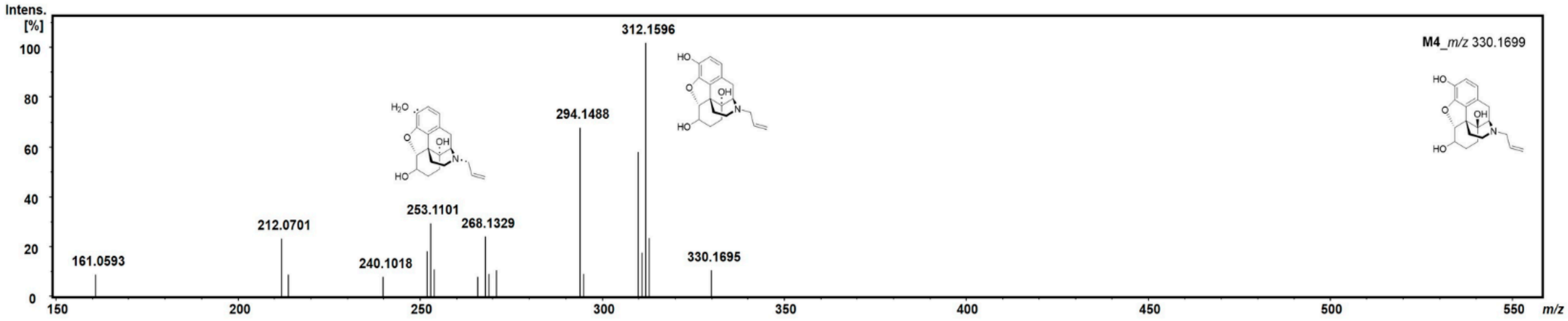

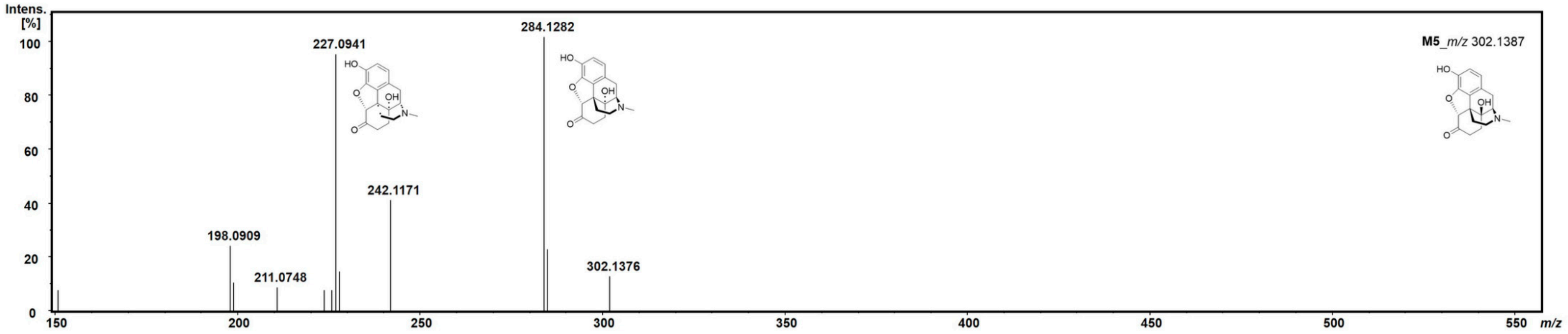

Figure S14. *Continued.*

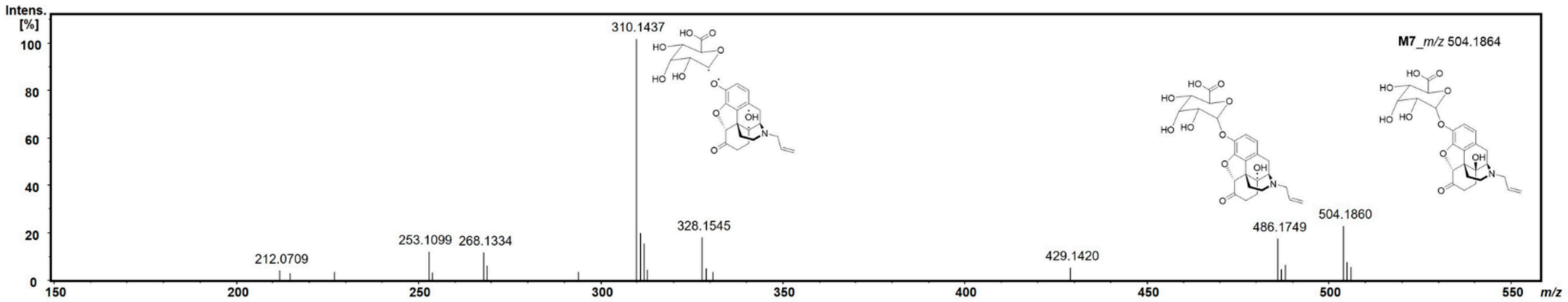

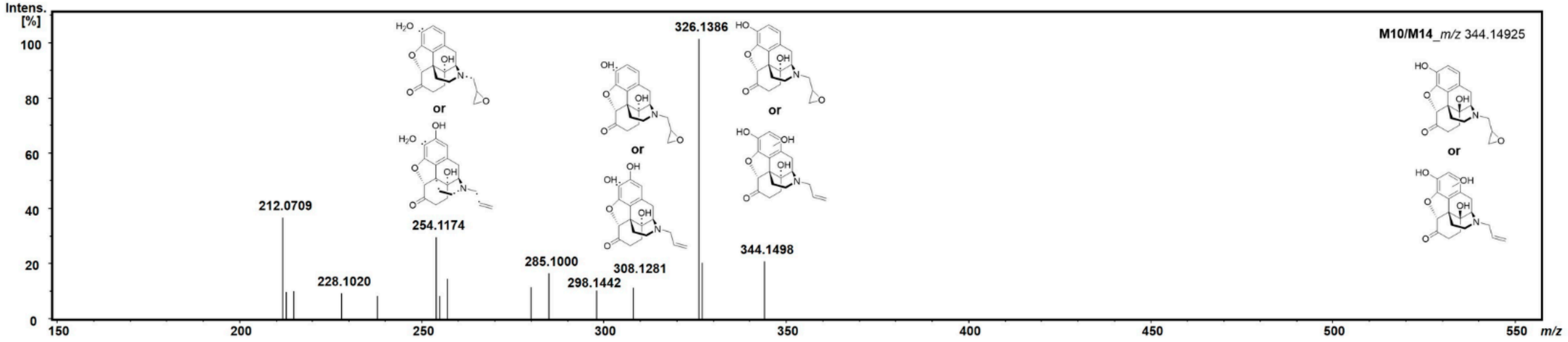

Figure S14. *Continued.*

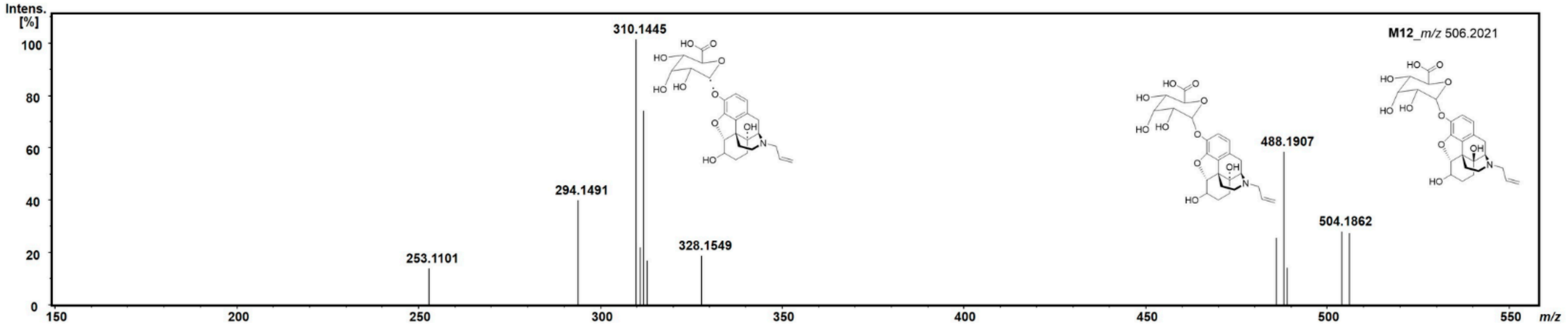

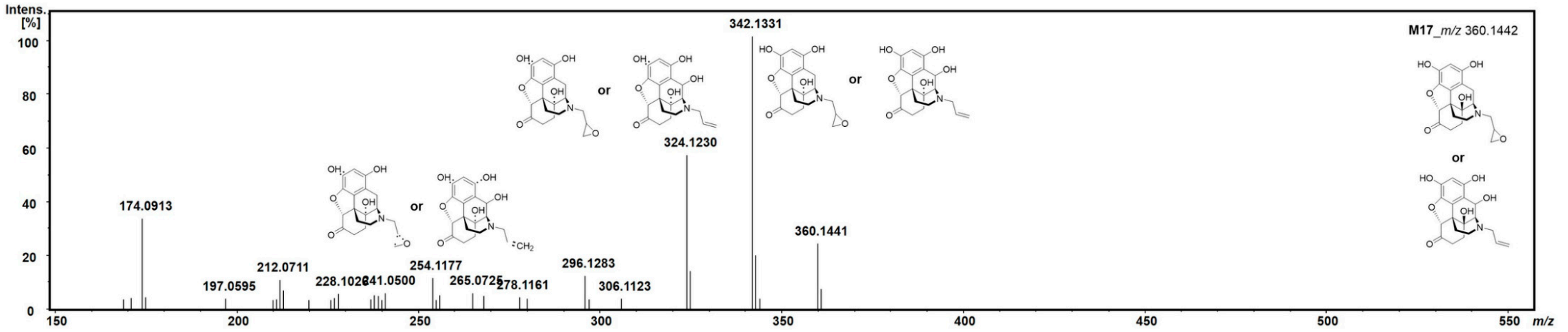

Figure S14. *Continued.*

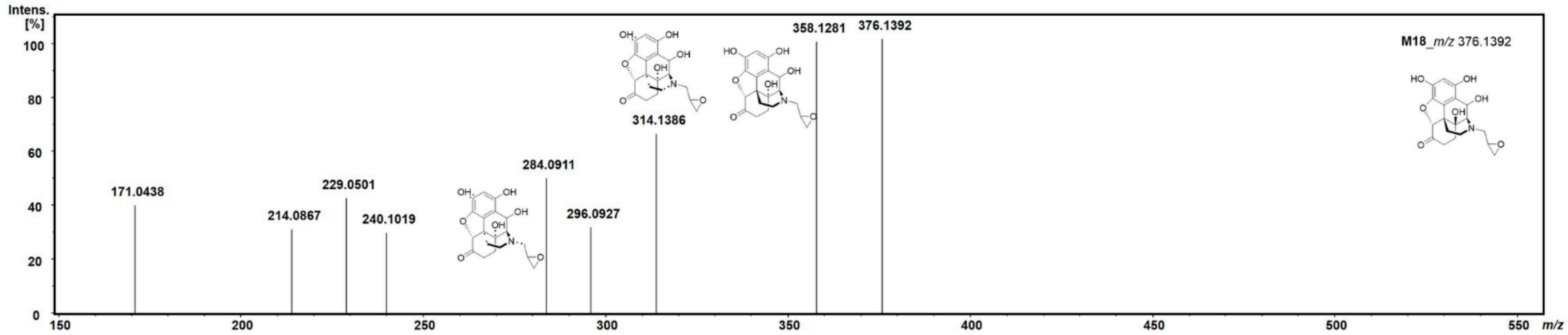

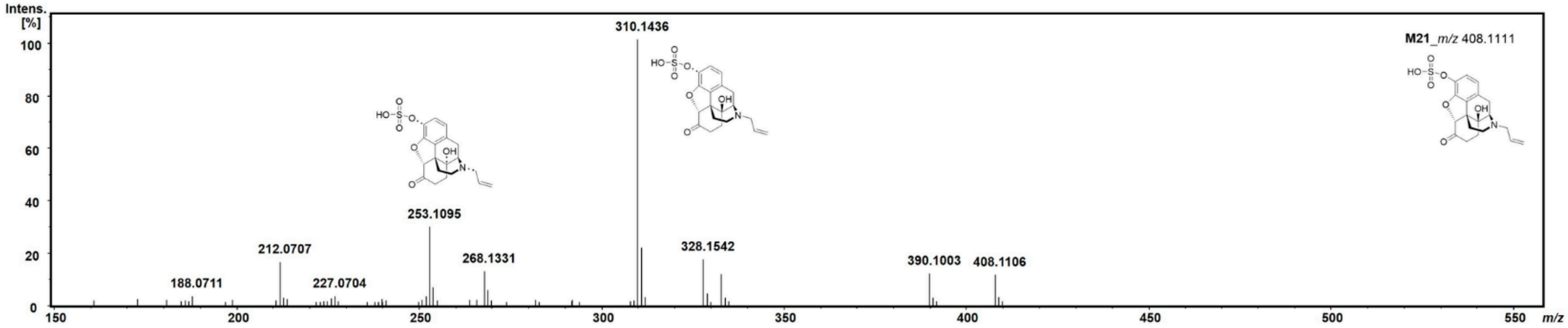

Figure S14. *Continued.*

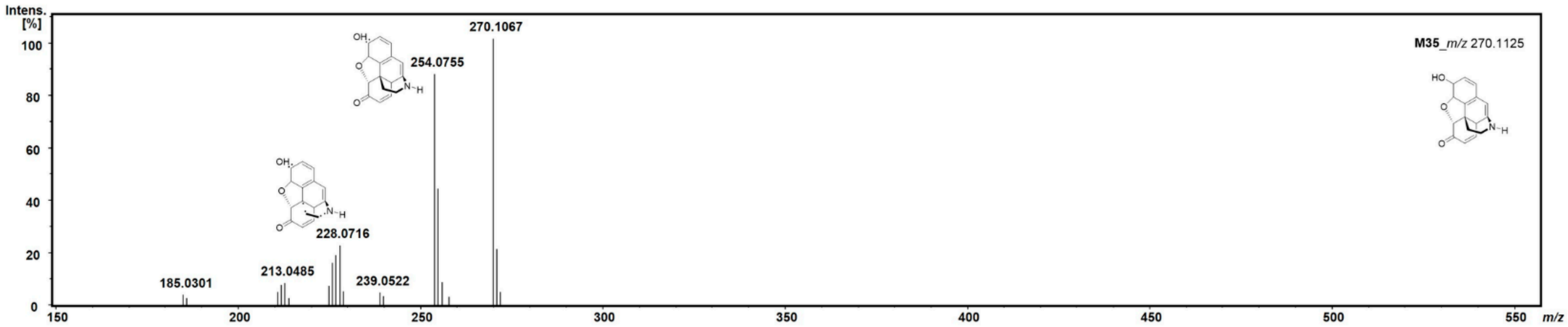

Figure S14. MS² spectra of naloxone and its ten metabolites detected in the exposed zebrafish larvae and the residual exposure medium, arranged by the labeling number in this study. The 13 metabolites were found in ZF larvae and residual exposure medium: M2, M4, M7, M12, M21, and M35 in the larvae, and M5, M10/M14, M17, and M18 in the medium. In the case of M10/M14 and M17, their MS² fragments were generated with two possible structures due to anticipating several structural isomers. The minor three metabolites (M8, M23, and M26) are not displayed here because their MS² spectra were not unavailable due to the low intensity of peaks.

Table S1. Effects on heartbeat rates in ZF larvae at 5 days post-fertilization (dpf) following 1 d and 5 d waterborne exposure, respectively, to varying concentrations of naloxone.

Concentration of naloxone (μM)	Relative heartbeat rates (%) of 5 dpf ZF larvae after 1 d waterborne exposure	Relative heartbeat rates (%) of 5 dpf ZF larvae after 5 d waterborne exposure
0.01	98 ± 2.9^{ns}	95 ± 2.8***
0.1	96 ± 3.4^{ns}	98 ± 4.1***
1.0	94 ± 2.1*	100 ± 1.6***
10	96 ± 4.0**	98 ± 2.6***
30	94 ± 4.2*	97 ± 3.0***
50	92 ± 1.4**	91 ± 3.3***
80	93 ± 3.8*	93 ± 4.5***
100	93 ± 2.6*	98 ± 3.0***
300	91 ± 4.6**	-
500	87 ± 2.1***	-

Heartbeat was measured as beats per minute (BPM) using DanioScope software, version 1.2.206. Relative heartbeat rates (%) were calculated in comparison to the vehicle (1% DMSO, *v*/*v*) treated control group which was set to 100%. All data are represented as mean ± standard error of the mean (s.e.m.) (n = 8-13 for 1 d exposure groups, n = 10–12 for 5 d exposure groups). The p-values were calculated by one-way ANOVA ($^{ns}p > 0.05$, $^{*}p < 0.05$, $^{**}p < 0.01$, $^{***}p < 0.001$) in comparison to the respective control group.

Table S2. Detailed information on naloxone and its metabolites identified from human biosamples and animal models (published data) and data of additional metabolite candidates from zebrafish larvae and HepaRG cells (this study).

Compounds	Metabolic reaction	Calculated exact masses (*m*/*z*)	Human [1, 5–13]		Rat [1–5]	Rabbit, Chicken [5]	Dog [1, 15]	Data from this study		
								HepaRG In Vitro Models [100 µM, 300 µM]	Zebrafish larvae [300 µM]	
			Plasma	Urine	Plasma, urine, feces	Urine	Urine		Larvae	Medium
Naloxone	Unchanged form	328.1543	+	+	+	+	+	+	+	+
M1	*N*-hydroxylation	345.1571								
M2	*N*-dealkylation (nornaloxone)	288.1230	+	+	+			+	+	+
M3	*N*-dealkylation + hydroxylation	304.1179								
M4	*N*-allyl-7,8-dihydro-14-hydroxynormorphine (naloxol)	330.1700	+	+	+	+	+	+	+[c]	
M5	*N*-CH_3 (*N*-dealkylation; oxymorphone)	302.1387								+[b]
M6	*N*-CH_2CH_3 (*N*-dealkylation)	316.1543								
M7	Glucuronidation	504.1864	+	+	+	+	+		+	+
M8	*N*-oxide + glucuronidation	520.1813							+[c]	
M9	Glucuronidation of M2	464.1551			+					
M10	*N*-oxidation	344.1492								+[a,b]
M11	Glucuronidation of M3	480.1500								
M12	Glucuronidation of M4	506.2021			+	+[d]			+[c]	
M13	2*glucuronidation of M4	682.2342								
M14	Epoxide formation<br>Hydroxylation in the benzene ring	344.1492								+[a,b]
M15	Epoxidation in alkene group of M4	346.1649								
M16	Epoxidation + methylation of M5	359.1727								
M17	Dihydroxylation	360.1442						+[e]		+[b]

	Hydroxylation + epoxide formation<br>*N*-oxide + epoxide formation									
M18	Dihydroxylation + epoxidation	376.1391								+[b]
M19	Glucuronidation of M14	536.1763								
M20	2*glucuronidation	680.2185								
M21	Sulfation	408.111			+			+	+	+
M22	2*sulfation	488.0680								
M23	Hydroxylation + methoxylation in benzene ring	358.1649							+[c]	
M24	Glucuronidation of M23	534.1970								
M25	Hydroxylation in alkene group	332.1492								
M26	Carboxylation in alkene group	346.1285								+[b]
M27	Naloxegol	652.3691								
M28	Addition of *S*-methyl	374.1421								
M29	Glutathione conjugate	633.2225								
M30	Cysteine conjugate	447.1584								
M31	Di-hydrogenation with the cleavage of carbon-oxygen bond in cyclopentane moiety	332.1856								
M32	Di-hydrogenation of M7with the cleavage of carbon-oxygen bond in cyclopentane moiety<br>Glucuronidation of M31	508.2177								
M33	Glucuronidation of M32	684.2498								
M34	2 hydroxylation in benzene ring and alkene part	348.1442								
M35	Rearrangement after elimination of hydroxyl group of M2	270.1125			+				+[c]	
M36	*N*-CH_2CH_3 of M4	318.1700								
M37	Glucuronidation of M1	521.1892								
	Total number of detected metabolites		3	3	7	3	2	4	8	8

All data from human samples and animal models were taken from published studies; Human plasma data were quoted from [8, 11–13] and urine data from [1, 5–10]. Rat plasma data were cited from [1–3] and urine and feces data from [1, 4, 8]. [a] The structural isomers M10 and M14 co-eluted in the LC-HRMS/MS setup used in this study, and accordingly, isomers were counted and quantified as one metabolite although they derive from different metabolic reactions (see Figure 2); [b] the metabolites were only detected in the surrounding exposure medium and not from extracted ZF larvae; [c] the metabolites were detected only in extracted ZF larvae and not in the surrounding exposure medium; [d] the metabolite was not detected in rabbit; [e] the metabolite was only detected at the higher exposure concentration of 300 µM naloxone. +: Peak detected.

Table S3. Mass list of naloxone and its metabolites used for LC-HRMS/MS and MALDI-FT-ICR measurements, respectively.

Compounds	Major ion species	
	$[M+H]^+$ in LC-HRMS/MS	$[M+K]^+$ in MALDI-FT-ICR
Naloxone	328.1543	366.110
M1	345.1571	383.113
M2	288.1230	326.078
M3	304.1179	342.073
M4	330.1700	368.125
M5	302.1387	340.094
M6	316.1543	354.110
M7	504.1864	542.142
M8	520.1813	558.137
M9	464.1551	502.111
M10/M14	344.1492	382.105
M11	480.1500	518.105
M12	506.2021	544.158
M13	682.2342	720.190
M15	346.1649	384.120
M16	359.1727	397.128
M17	360.1442	398.100
M18	376.1391	414.095
M19	536.1763	574.132
M20	680.2185	718.174
M21	408.1111	446.067
M22	488.0680	526.023
M23	358.1649	396.120
M24	534.1970	572.152
M25	332.1492	370.105
M26	346.1285	384.084
M27	652.3691	690.325
M28	374.1421	412.097
M29	633.2225	671.178
M30	447.1584	485.114
M31	332.1856	370.141
M32	508.2177	546.173
M33	684.2498	722.205

M34	348.1442	386.100
M35	270.1125	308.068
M36	318.1700	356.125
M37	521.1892	559.145

Table S4. Detailed experimental conditions of MSI sample preparation using ZF larvae homogenates evaluated in this study.

Steps	Conditions	Variable parameters
1) Homogenate block*	Gelatin medium as lower sample layer**	40% (*w*/*v*), 30% (*w*/*v*)
2) Cryosectioning	Thickness***	10 μm, (15 μm, 20 μm)
3) Matrix deposition****	Matrix reagent	15 mg mL^{-1}, 30 mg mL^{-1} (for DHB), 5 mg mL^{-1}, 15 mg mL^{-1} (for CHCA)
	Addition of TFA	0.1%, 0.5%, 1.0% (for DHB), 0.1% (for CHCA)
	Spray deposition passes	14 passes, 28 passes (for DHB), 8 passes, 14 passes (for CHCA)

The steps are listed in order of sample preparation for MSI. Conditions of our previously used protocol [14, 16] are highlighted in bold. *The homogenate block is composed of a lower layer (the same thickness as the tested larvae section) of gelatin medium used as a blank layer for spiking ZF homogenates on top. **30% gelatin medium was additionally tested in this study since only 40% gelatin was tested for embedding a whole body of ZF larva in previous studies [14, 16]. ***For cryosectioning, 15 μm and 20 μm thickness were also tested, however, these data are not shown due to many practical failures in the following matrix deposition step. Thus, all presented data are from 10 μm sections. ****The detailed deposition condition for each matrix reagent (DHB and CHCA) is described in the Mass Spectrometry Image Analysis of ZF Larva by MALDI-FT-ICR. DHB: 2,5-Dihydroxybenzoic acid, CHCA: α-Cyano-4-hydroxycinnamic acid, TFA; Trifluoroacetic acid.

References

[1] Kaleo, I.B.D. Joint Meeting of the Anesthetic and Analgesic Drug Products and the Drug Safety and Risk Management Advisory Committees October 5, 2016: Advisory Committee Briefing Materials, **2016**.

[2] Joshi, A.; Parris, B.; Liu, Y.; Heidbreder, C.; Gerk, P.M.; Halquist, M. Quantitative determination of buprenorphine, naloxone and their metabolites in rat plasma using hydrophilic interaction liquid chromatography coupled with tandem mass spectrometry. *Biomedical chromatography : BMC*, **2017**, *31* (2). DOI: 10.1002/bmc.3785.

[3] Committee for Medicinal Products for Human Use (CHMP), European Medicines Agency. *Assessment report: Nyxoid*, **September 14, 2017**.

[4] Misra, A.L.; Pontani, R.B.; Vadlamani, N.L.; Mulé, S.J. Physiological disposition and biotransformation of allyl-1', 3' - 14C naloxone in the rat and some comparative observations on nalorphine. *The Journal of pharmacology and experimental therapeutics*, **1976**, *196*, 257–268.

[5] Fujimoto, J.M. Isolation of naloxone-3-glucuronide from human urine. *Proceedings of the Society for Experimental Biology and Medicine. Society for Experimental Biology and Medicine (New York, N.Y.)*, **1970**, *133* (1), 317–319. DOI: 10.1002/jps.2600601030.

[6] S. H. Weinstein, M. Pfeffer, J. M. Schor, L. Indindoli, M. Mintz. Metabolites of naloxone in human urine. *Journal of Pharmaceutical Sciences*, **1971**, 60 (10), 1567–1568. DOI: 10.1002/jps.2600601030.

[7] van Dorp, E.L.A.; Yassen, A.; Dahan, A. Naloxone treatment in opioid addiction: the risks and benefits. *Expert opinion on drug safety*, **2007**, *6* (2), 125–132. DOI: 10.1517/14740338.6.2.125.

[8] FDA Advisory Committee on the Most Appropriate Dose or Doses of Naloxone to Reverse the Effects of Life-threatening Opioid Overdose in the Community Settings: ADVISORY COMMITTEE BRIEFING MATERIALS. *Joint Meeting of the Anesthetic and Analgesic Drug Products Advisory Committee and the Drug Safety and Risk Management Advisory Committee on October 5, 2016*, **2016**.

[9] Rzasa Lynn, R.; Galinkin, J.L. Naloxone dosage for opioid reversal: current evidence and clinical implications. *Therapeutic advances in drug safety*, **2018**, *9* (1), 63–88. DOI: 10.1177/2042098617744161.

[10] Haidari, M.; Mansani, S.; Ponds, D.; Romero, L.; Alsaab, S. Consumption of Movantik™ (Naloxegol) results in detection of naloxone in the patient's urine evaluated by confirmatory urine drug testing. *Clinical biochemistry*, **2019**, *67*, 48–53. DOI: 10.1016/j.clinbiochem.2019.03.006.

[11] CHIANG, C. Pharmacokinetics of the combination tablet of buprenorphine and naloxone. *Drug and alcohol dependence*, **2003**, *70*, S39-S47.

[12] Wiegand, S.L.; Swortwood, M.J.; Huestis, M.A.; Thorp, J.; Jones, H.E.; Vora, N.L. Naloxone and Metabolites Quantification in Cord Blood of Prenatally Exposed Newborns and Correlations with Maternal Concentrations. *AJP reports*, **2016**, *6*, e385-e390.

[13] Swortwood, M.J.; Scheidweiler, K.B.; Barnes, A.J.; Jansson, L.M.; Huestis, M.A. Simultaneous quantification of buprenorphine, naloxone and phase I and II metabolites in plasma and breastmilk by liquid chromatography-tandem mass spectrometry. *Journal of chromatography. A*, **2016**, *1446*, 70–77.

[14] Park, Y.M.; Meyer, M.R.; Müller, R.; Herrmann, J. Drug Administration Routes Impact the Metabolism of a Synthetic Cannabinoid in the Zebrafish Larvae Model. *Molecules (Basel, Switzerland)*, **2020**, *25* (19), DOI: 10.3390/molecules25194474.

[15] Garrett, E.R.; Shyu, W.C.; Ulubelen, A. Pharmacokinetics of morphine and its surrogates. VIII: Naloxone and naloxone conjugate pharmacokinetics in dogs as a function of dose and as affected by simultaneously administered morphine. *Journal of Pharmaceutical Sciences*, **1986**, *75*, 1127–1136.

[16] Park, Y.M.; Dahlem, C.; Meyer, M.R.; Kiemer, A.K.; Müller, R.; Herrmann, J. Induction of Liver Size Reduction in Zebrafish Larvae by the Emerging Synthetic Cannabinoid 4F-MDMB-BINACA and Its Impact on Drug Metabolism. *Molecules (Basel, Switzerland)*, **2022**, 27 (4), 1290. DOI: 10.3390/molecules27041290.
